# Boosting Nitrogen Reduction Reaction via Electronic Coupling of Atomically Dispersed Bismuth with Titanium Nitride Nanorods

**DOI:** 10.1002/advs.202104245

**Published:** 2021-12-02

**Authors:** Zichao Xi, Ke Shi, Xuan Xu, Peng Jing, Baocang Liu, Rui Gao, Jun Zhang

**Affiliations:** ^1^ School of Chemistry and Chemical Engineering Inner Mongolia Engineering and Technology Research Center for Catalytic Conversion and Utilization of Carbon Resource Molecules and Inner Mongolia Key Lab of Nanoscience and Nanotechnology Inner Mongolia University 235 West University Street Hohhot 010021 P. R. China

**Keywords:** density functional theory calculation, electrocatalysis, monolithic electrodes, nitrogen reduction reaction, single bismuth atoms

## Abstract

Electrocatalytic nitrogen reduction reaction (NRR) is a promising alternative to the traditional Haber–Bosch process. However, the sluggish kinetics and competitive hydrogen evolution reaction result in poor NH_3_ yield and low Faradaic efficiency (FE). Herein, single bismuth atoms incorporated hollow titanium nitride nanorods encapsulated in nitrogen‐doped carbon layer (NC) supported on carbon cloth (NC/Bi SAs/TiN/CC) is constructed for electrocatalytic NRR. Impressively, as an integrated electrode, it exhibits a superior ammonia yield rate of 76.15 µg mg_cat_^−1^ h^−1^ (9859 µg μmol_Bi_^−1^ h^−1^) at −0.8 V versus RHE and a high FE of 24.60% at −0.5 V versus RHE in 0.1 m Na_2_SO_4_ solution, which can retain stable performance in 10 h continuous operation, surpassing the overwhelming majority of reported Bi‐based NRR catalysts. Coupling various characterizations with theory calculations, it is disclosed that the unique monolithic core‐shell configuration with porous structure endows abundant accessible active sites, outstanding charge‐transfer property, and good stability, while the cooperation effect of Bi SAs and TiN can simultaneously promote the hydrogenation of N_2_ into NH_3_* on the TiN surface and the desorption of NH_3_^*^ to release NH_3_ on the Bi SA sites. These features result in the significant promotion of NRR performance.

## Graphical abstract

A novel electrode of bismuth single‐atom (Bi SA) incorporated hollow titanium nitride nanorods encapsulated in nitrogen‐doped carbon layer on carbon cloth is designed for nitrogen reduction reaction (NRR). The electronic coupling of Bi SA and TiN promotes the hydrogenation of N_2_ into NH_3_^*^ on the TiN and the desorption of NH_3_^*^ from single‐atomic Bi sites, therefore boosting the NRR performance.

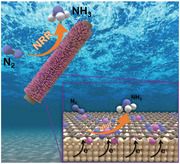

## 1 Introduction

For a long time, ammonia is always regarded as a nitrogen‐rich source of fertilizer and a promising energy storage carrier, as well as a raw material for many chemical products.^[^ ^1^, ^2^ ^]^ To date, industrial ammonia production mainly relies on the traditional Haber‐Bosch process, in which high purity nitrogen and hydrogen react at high temperature and pressure (400–600 °C, 20–40 MPa).^[^ ^3^ ^]^ However, the harsh reaction conditions result in low efficiency, high energy consumption, and greenhouse gas emission.^[^ ^4^ ^]^ With this regard, nitrogen reduction reaction (NRR) at room temperature and atmospheric pressure is a promising alternative but faces a major challenge of breaking the N≡N bond with the strong bond energy of 940.95 kJ mol^−1^.^[^ ^3^ ^]^ Electrocatalytic NRR happening at ambient conditions provides a feasible direction and thus becomes a hot research topic.^[^ ^5^, ^6^ ^]^ During electrocatalytic NRR, competitive hydrogen evolution reaction (HER) takes place simultaneously, which results in poor NH_3_ yield and low Faradaic efficiency (FE), and consequently hinders its large‐scale applications.^[^ ^7^ ^]^ Therefore, electrocatalysts with high efficiency and high selectivity are highly demanded for nitrogen neduction reaction (NRR).

Noble‐metal catalysts, such as Au,^[^ ^8^ ^]^ Pd,^[^ ^9^ ^]^ Ru,^[^ ^10^ ^]^ and Rh^[^ ^11^ ^]^ exhibit favorable activity in NRR, but their practical applicability is severely restricted by their scarcity and high cost. As a low‐cost and environmentally‐benign semimetal, Bi, especially Bi at the nanoscale, is a potential NRR candidate because it has limited surface electron accessibility, high p‐electron donating power, and weak bonding with H atoms,^[^ ^12^, ^13^, ^14^, ^15^, ^16^, ^17^, ^18^ ^]^ which not only facilitates to N_2_ adsorption and activation but also can suppress the competitive HER. In the last few years, various Bi‐based materials with nanostructure, including Bi nanosheets,^[^ ^16^, ^19^ ^]^ defect‐Bi nanoplates,^[^ ^15^ ^]^ Bi nanodendrites,^[^ ^20^ ^]^ Bi nanoarrays,^[^ ^21^ ^]^ Bi nanocrystals,^[^ ^13^ ^]^ densely packed Bi nanoparticles,^[^ ^14^ ^]^ ultrathin porous Bi_5_O_7_I nanotubes,^[^ ^22^ ^]^ S—Bi nanobelts,^[^ ^23^ ^]^ and flower‐like *β*‐Bi_2_O_3_^[^ ^12^ ^]^ have been explored as active catalysts for NRR. However, the NRR performance of the reported Bi‐based materials is still limited due to their poor electrical conductivity, insufficient active sites, as well as self‐aggregation problems.^[^ ^15^, ^16^, ^24^ ^]^ Therefore, exploring Bi‐based composite NRR catalysts with superior electronic conductivity, highly exposed active sites, ideal electronic structure, and good stability by rational structure and composite design to improve the electrocatalytic NRR performance is of great significance but still remains a challenge.

Recently, single‐atom catalysts (SACs) have become an emerging field in catalytic science owing to their unique electronic structure, uniform distribution in the substrate, maximized atomic utilization (close to 100%), and unsaturated active sites.^[^ ^25^, ^26^ ^]^ These specific characteristics make SACs achieve superior performance in many reactions, for example, HER,^[^ ^27^ ^]^ oxygen evolution reaction (OER),^[^ ^28^ ^]^ oxygen reduction reaction (ORR),^[^ ^29^ ^]^ carbon dioxide reduction reaction (CO_2_RR),^[^ ^30^, ^31^ ^]^ and NRR.^[^ ^3^ ^]^ Although many SACs, such as Mo,^[^ ^32^ ^]^ Ru,^[^ ^10^ ^]^ Fe,^[^ ^33^ ^]^ Y,^[^ ^34^ ^]^ and Sc,^[^ ^34^ ^]^ with enhanced catalytic performance have been used in NRR, there is no report of single Bi atoms for catalyzing NRR. In addition, these reported SACs are normally only available as powders, which is not convenient for making electrodes. The polymer binder (e.g., Nafion) is commonly used for attaching the electrocatalysts to the collectors, which largely increases the charge transfer resistance, blocks the active sites, and impedes the diffusion of electrolyte, and therefore reduces the overall catalytic activity and stability of the electrocatalysts.^[^ ^35^, ^36^ ^]^ Thus, developing an efficient strategy to directly grow single Bi atoms on various substrates and fabricate 3D self‐supported Bi SAs‐based monolithic electrodes with abundant active sites, reduced charge transport resistance, and improved HER performance is highly demanded but still hard to be realized.

In the work, we explore a simple and efficient avenue to construct a 3D self‐standing integrated electrode consisting of single Bi atoms incorporated hollow titanium nitride nanorods encapsulated in single Bi atoms anchored nitrogen‐doped carbon layer (NC/Bi SAs/TiN) with hierarchical porosity on carbon cloth (CC) for efficient NRR. In such NC/Bi SAs/TiN/CC electrode, the 3D opened porous structure ensures the sufficient exposure of active sites and the efficient mass transfer, which is conducive to improving the catalyst activity, while the 3D self‐supported monolithic configuration can enhance the catalytic stability. Meanwhile, the confined effect of NC matrix and TiN nanorods not only helps to prevent the Bi SAs from corrosion and aggregation but also facilitates the charge transport in the process of electrocatalytic reaction. More importantly, the cooperative effect between Bi SAs and TiN substrate simultaneously promotes the hydrogenation of N_2_ molecule into NH_3_^*^ on the TiN substrate and the desorption of NH_3_^*^ from single‐atomic Bi sites to release NH_3_, and resulting in an enhanced NRR activity. As a result, the NC/Bi SAs/TiN/CC electrode achieves a superior NH_3_ yield rate of 76.15 µg mg_cat_^−1^ h^−1^ (9859 µg μmol_Bi_^−1^ h^−1^) at −0.8 V versus RHE and a high FE of 24.60% at −0.5 V versus RHE in 0.1 m Na_2_SO_4_ solution under ambient conditions.

## 2 Results and Discussion

**Scheme 1:**
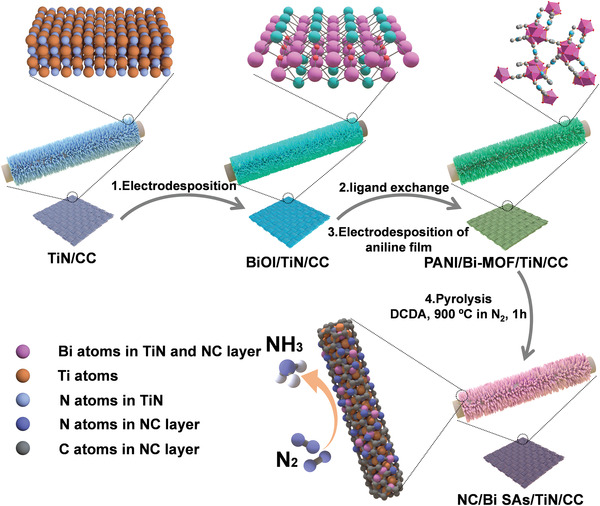
Schematic illustration of the synthetic process of the NC/Bi SAs/TiN/CC.

The NC/Bi SAs/TiN/CC electrocatalyst was synthesized by a four‐step process (**Scheme** **1**). Initially, the BiOI nanosheets were grown on TiN/CC via electrodeposition method. Subsequently, the BiOI/TiN/CC was in situ transformed to Bi‐MOF/TiN/CC by a novel hydrothermal ligand exchange method. Then, a layer of polyaniline (PANI) was electrodeposited on the surface of Bi‐MOF/TiN/CC. Finally, the PANI/Bi‐MOF/TiN/CC was converted to NC/Bi SAs/TiN/CC via a pyrolysis process in the presence of dicyandiamide (DCDA) under N_2_ atmosphere. The Bi loading on NC/Bi SAs/TiN/CC is measured to be 0.023 mg cm^−2^ by inductively coupled plasma‐atomic emission spectroscopy (ICP‐AES) (Table S1, Supporting Information). The PANI layer plays a pivotal role in simultaneously forming single Bi atoms incorporated TiN nanorods and NC layers. It not only affords C and N sources to form NC layers to prevent the loss of Bi species but also helps to retain more single Bi atoms on the surface of TiN nanorods as almost no Bi species are detected in the sample fabricated via the similar approach but without PANI layer protection (Figure S1, Supporting Information). In addition, the decomposition of DCDA could release NH_3_, which promotes the atomization of Bi SAs and the doping of N in carbon layers.^[^ ^31^ ^]^

**Figure 1:**
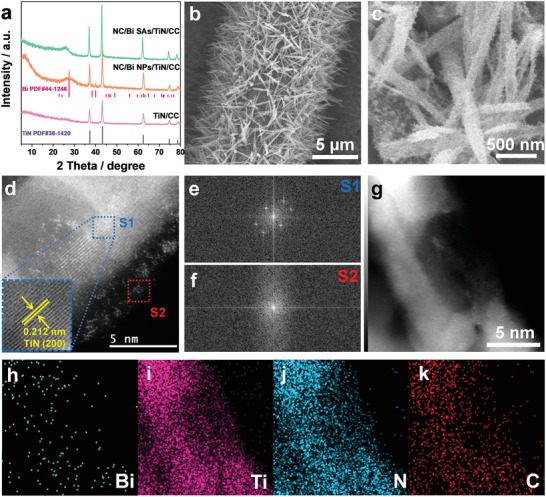
a) The XRD patterns of NC/Bi SAs/TiN/CC, NC/Bi NPs/TiN/CC, and TiN/CC; b) Low‐ and c) High‐resolution SEM images of NC/Bi SAs/TiN/CC; d) AC‐HAADF‐STEM image of NC/Bi SAs/TiN/CC (the bright dots in image represent single Bi atoms); e,f) Corresponding FFT patterns of regions S1 and S2; g) STEM image of NC/Bi SAs/TiN/CC; h–k) Corresponding elemental mappings of Bi, Ti, N, and C in NC/Bi SAs/TiN/CC.

The crystalline phase after each reaction step was monitored by X‐ray powder diffraction (XRD). It is seen that the TiN/CC displays the diffraction peaks at around 36.66°, 42.59°, 61.81°, 74.07°, and 77.96° ascribed to the (111), (200), (220), (311), and (222) planes of cubic TiN (PDF no. 38‐1420) (**Figure** **1**a), suggesting the successful growth of TiN nanorods on CC. After the electrodeposition of BiOI nanosheets, the main diffraction peaks at 29.6, 31.7, and 55.2° appear, which are associated with the (102), (110), and (212) planes of face‐centered BiOI (PDF no. 10‐0445) (Figure S2, Supporting Information). Following the ligand exchange reaction in the presence of trimesic acid, the diffraction peaks attributed to CAU‐17 [Bi(BTC)(H_2_O)] are detected,^[^ ^14^ ^]^ implying the transformation of BiOI nanosheets into Bi‐MOF. After further electrodeposition of PANI layer, the diffraction peaks of Bi‐MOF with reduced intensity are still reserved, suggesting the successful coating of PANI layer. As for the NC/Bi NPs/TiN/CC electrode, besides the characteristic peaks of TiN, the additional diffraction peaks at 27.2°, 37.9°, and 39.6° associated with the (012), (104), and (110) planes of hexagonal metallic bismuth (PDF no. 44‐1246) are observed, indicating that the Bi nanoparticles have been loaded on TiN nanorods. Unexpectedly, in the NC/Bi SAs/TiN/CC electrode, only the characteristic diffraction peaks of TiN (PDF no. 38‐1420) can be detected while the characteristic diffraction peaks of Bi disappear, revealing the presence of highly dispersed Bi species on TiN surface. Additionally, the board peak at around 26.5°, corresponding to the (002) plane of graphitic carbon, can be found in NC/Bi NPs/TiN/CC and NC/Bi SAs/TiN/CC. This result confirms the existence of graphitic carbon layer in NC/Bi NPs/TiN/CC and NC/Bi SAs/TiN/CC. XRD patterns of NC/TiN/CC and TiN/CC only show the diffraction peaks of cubic TiN (PDF no. 38‐1420) (Figure S3, Supporting Information). No characteristic peaks associated with Bi species are detected in the XRD pattern of NC/Bi SAs/CC, implying that the existed Bi species may be atomically dispersed.

The morphology evolution after each reaction step is also monitored by the scanning electron microscopy (SEM) (Figures S4–S6, Supporting Information and Figure 1b). Obviously, the as‐synthesized TiN on CC exhibits a porous nanorod‐like structure with a mean length of ≈3.5 µm and width of ≈100 nm (Figure S4, Supporting Information). SEM images of BiOI/TiN/CC indicate that the interconnected BiOI nanosheets with a lateral size of 1.5–2.5 µm and a thickness of ≈50 nm have been vertically grown on TiN surface (Figure S5, Supporting Information). After ligand exchange, the BiOI nanosheets are in situ converted into Bi‐MOF nanoribbons with an average width of ≈2.5 µm and a thickness of ≈650 nm (Figure S6a, Supporting Information). In comparison to the Bi‐MOF/TiN/CC, the PANI/Bi‐MOF/TiN/CC exhibits a much rough surface (Figure S6b, Supporting Information), indicating that PANI layer has been successfully coated on the surface of Bi‐MOF. Interestingly, upon the pyrolysis of PANI/Bi‐MOF/TiN/CC in the presence of DCDA under N_2_ atmosphere, the uniform NC/Bi SAs/TiN/CC composite nanorods (≈3 µm in mean length and ≈180 nm average width) with rough surface on CC are achieved (Figure 1b,c). This result implies that the PANI/Bi‐MOF nanobelt has been converted to Bi incorporated NC layer that is tightly coated on the surface of TiN nanorods. The aberration‐corrected high‐angle annular dark‐field scanning transmission electron microscopy (AC‐HAADF‐STEM) images reveal that the isolated bright spots corresponding to single Bi atoms are well dispersed on both NC layer and TiN nanorod without agglomeration in the NC/Bi SAs/TiN/CC (Figure 1d and Figure S7, Supporting Information). Meanwhile, the bright nanorod is surrounded by gray border, verifying that the TiN nanorod is wrapped by an NC layer with a thickness of ≈2 nm. In addition, the interplanar distance of 0.212 nm is clearly discerned in NC/Bi SAs/TiN/CC, matching well with the (200) plane of TiN. The corresponding fast Fourier transform (FFT) patterns of regions S1 and S2 in Figure 1d demonstrate the amorphous nature of Bi SAs in NC layer and monocrystalline feature of TiN nanorod. The corresponding elemental mapping images show that the Ti, N, and C elements are homogeneously distributed throughout the whole NC/Bi SAs/TiN/CC composite nanorod while the Bi element is dispersed discretely on the composite nanorod. In the light of above microscopic characterizations, it is confirmed that the TiN nanorod is tightly encapsulated by an NC layer and Bi SAs are simultaneously dispersed on the NC layer and TiN nanorod in NC/Bi SAs/TiN/CC. Differently, although the NC/Bi NPs/TiN exhibits a porous nanorod‐like structure, besides the lattice spacing of 0.244 nm corresponding to the (111) plane of TiN, the interplanar distance of 0.155 nm matching well with the (107) plane of Bi is clearly discerned in NC/Bi NPs/TiN (Figure S8, Supporting Information). Meanwhile, around the TiN nanorod and Bi nanoparticles, the few‐layered carbon layer is found (Figure S8b, Supporting Information). These results confirm that the Bi NPs supported TiN nanorod is encapsulated by NC layer in NC/Bi NPs/TiN composite nanorods. The corresponding EDX elemental mappings (Figure S9, Supporting Information) show the homogeneous distribution of C, N, Ti, and Bi elements on the NC/Bi NPs/TiN composite nanorod.

**Figure 2:**
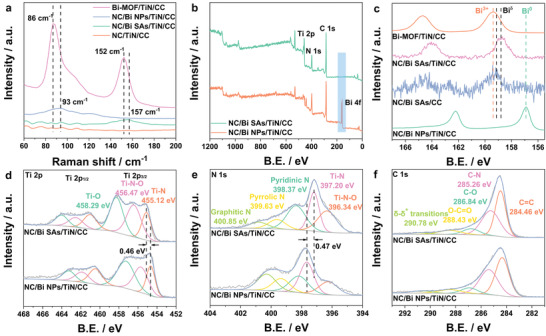
a) Raman spectra of Bi‐MOF/TiN/CC, NC/Bi NPs/TiN/CC, NC/Bi SAs/TiN/CC, and NC/TiN/CC and XPS spectra of b) survey, c) Bi 4f, d) Ti 2p, e) N 1s, and f) C 1s for NC/Bi SAs/TiN/CC and NC/Bi NPs/TiN/CC.

Raman spectra of Bi‐MOF/TiN/CC, NC/Bi SAs/TiN/CC, and NC/Bi NPs/TiN/CC were measured to explore the chemical conversion of Bi species in various stages. Two characteristic peaks at 86 and 152 cm^−1^ indexed to the Bi‐MOF^[^ ^14^ ^]^ are observed in the Bi‐MOF/TiN/CC precursor, suggesting the achievement of Bi‐MOF on TiN nanorods (**Figure** **2**a). After pyrolysis at 700 °C in the presence of DCDA under N_2_ atmosphere, the previous peaks disappear and a new peak at 93 cm^−1^ assigned to A_1g_ stretching mode of Bi—Bi bonds appears,^[^ ^13^, ^37^, ^38^ ^]^ proving that Bi‐MOF is completely converted to Bi nanoparticles in NC/Bi NPs/TiN/CC. Note that in the Raman spectroscopy of NC/Bi SAs/TiN/CC, the peak at 93 cm^−1^ associated with Bi nanoparticles vanishes, and a new broad peak at around 157 cm^−1^ that may originate from the stretching vibration of Bi‼N bonds emerges. Further combined with previous literature reports, it can be deduced that the Bi nanoparticles may be converted to single Bi atoms with BiN_4_ configuration in NC/Bi SAs/TiN/CC.^[^ ^31^ ^]^ As a contrast, no peaks associated with Bi species are detected in the NC/TiN/CC. In addition, Raman spectra of NC/Bi SAs/TiN/CC and NC/Bi NPs/TiN/CC also display two peaks at high wavenumbers of 1367 and 1599 cm^−1^ (Figure S10, Supporting Information), which are assigned to the D band and G band of graphitized carbon, respectively, further verifying the presence of carbon layer in NC/Bi SAs/TiN/CC and NC/Bi NPs/TiN/CC.

X‐ray photoelectron spectroscopy (XPS) measurements were performed to further explore the elemental composition and valence state of Bi‐MOFs/CC, NC/Bi SAs/TiN/CC, and NC/Bi NPs/TiN/CC. The survey scan spectra (Figure 2b) demonstrate that the strong peaks of C, N, O, Ti, and Bi elements are found in NC/Bi NPs/TiN/CC, while only weak signal of Bi is observed in NC/Bi SAs/TiN/CC, suggesting that the Bi content in NC/Bi SAs/TiN/CC is far less than that in NC/Bi NPs/TiN/CC. The high‐resolution Bi 4f spectra can be deconvoluted into two peaks at binding energies of 156.8–159.4 and 162.2–164.7 eV, corresponding to Bi 4f_7/2_ and Bi 4f_5/2_ orbitals, respectively. Obviously, the doublet in Bi 4f spectra for NC/Bi SAs/TiN/CC and NC/Bi SAs/CC locate at binding energies of 158.72 eV (4f_7/2_)/164.02 eV (4f_5/2_) and 159.23 eV (4f_7/2_)/164.09 eV (4f_5/2_), which are higher than that for NC/Bi NPs/TiN/CC, but lower than that for Bi‐MOFs/CC, suggesting the valance state of Bi atoms in NC/Bi SAs/TiN/CC and NC/Bi SAs/CC is between 0 and +3 (Bi^*δ*+^, 0 < *δ* < 3) (Figure 2c).^[^ ^31^ ^]^ The Bi^*δ*+^ should be associated with single Bi atoms.^[^ ^31^ ^]^ It is worth noting that the binding energy of Bi 4f in NC/Bi SAs/TiN/CC exhibits a negative shift of 0.46 eV compared to that in NC/Bi SAs/CC, implying a higher electron density around Bi SAs decorated on TiN. Additionally, the loading of Bi decreases significantly in NC/Bi SAs/CC without TiN support because the Bi 4f XPS spectra show that the signal of Bi in NC/Bi SAs/CC is obviously weaker than that in NC/Bi SAs/TiN/CC. This result indicates that the TiN substrate in the NC/Bi SAs/TiN/CC facilitates the anchoring of Bi SAs and prevents Bi from losing at high‐temperature. In Ti 2p XPS spectra, the deconvoluted spectra show three doublets at 454.67–455.12 eV/460.49–461.04 eV, 455.73–456.47 eV/461.91–462.55 eV, and 457.33–458.29 eV/463.30–464.06 eV, being assigned to Ti—N, Ti—N—O, and Ti—O, respectively.^[^ ^39^ ^]^ Notably, the binding energy of Ti 2p_3/2_ in NC/Bi SAs/TiN/CC has a positive shift of 0.46 eV in comparison with that in NC/Bi NPs/TiN/CC, further suggesting that more electrons transfer from Ti to Bi SAs that located on TiN in NC/Bi SAs/TiN/CC.^[^ ^40^, ^41^ ^]^ The deconvolution of XPS spectra for N 1s exhibit five peaks at 396.25–396.34, 397.20–397.67, 398.20–398.37, 399.35–399.63, and 400.30–400.85 eV, (Figure 2e) corresponding to N—Ti—O, TiN, pyridinic N, pyrrolic N, and graphitic N, respectively,^[^ ^42^ ^]^ which indicates the formation of N doped carbon layer and TiN. Furthermore, The N 1s peak of Ti—N bond in NC/Bi SAs/TiN/CC shows anegative shift, further suggesting an electron transfer from Bi SAs to N atoms in TiN.^[^ ^40^, ^41^ ^]^ The higher electron density around single Bi atoms is beneficial to participating in the hydrogenation step of NRR.^[^ ^43^ ^]^ The C 1s spectra of NC/Bi SAs/TiN/CC and NC/Bi SAs/TiN/CC (Figure 2f) show five characteristic peaks at 284.46, 285.26, 286.84, 288.43, and 290.78 eV attributed to C═C, C—N, C—O, O—C═O, and *δ*—*δ* ^*^ transitions, respectively, further demonstrating the successful doping of N atoms in carbon layer.^[^ ^44^ ^]^

**Figure 3:**
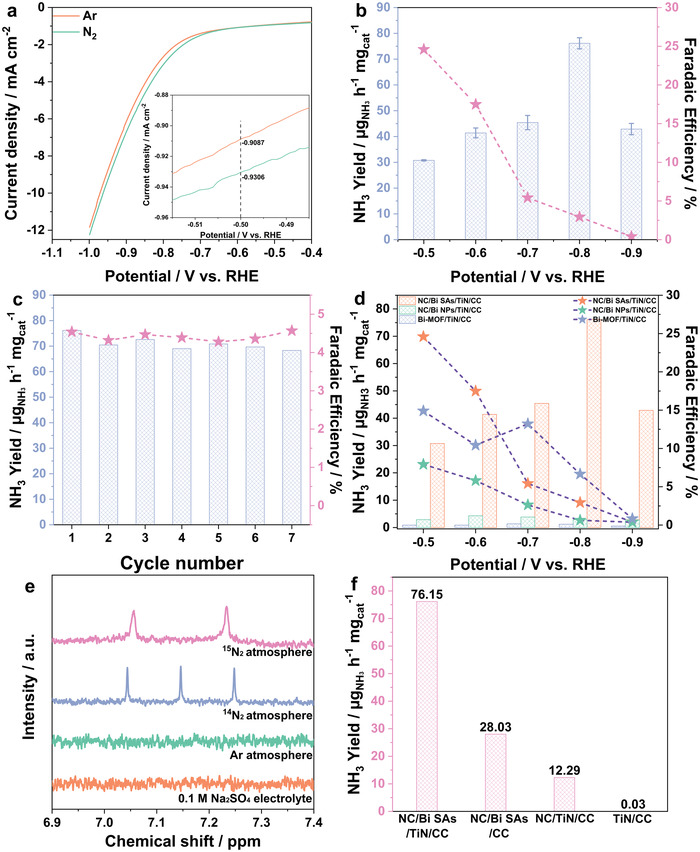
a) LSV curves of NC/Bi SAs/TiN/CC in 0.1 m N_2_‐ and Ar‐ saturated Na_2_SO_4_ electrolyte; b) NH_3_ formation rate and FE of NC/Bi SAs/TiN/CC at different potentials; c) Recycling stability tests on NC/Bi SAs/TiN/CC at 0.8 V versus RHE for 7 times; d) NH_3_ formation rate and FE of NC/Bi SAs/TiN/CC, NC/Bi NPs/TiN/CC, and Bi‐MOF/TiN/CC at different potentials; e) ^1^H NMR analysis of the blank electrolyte and the electrolyte fed by Ar, ^14^N_2_, and ^15^N_2_ after NRR reactions; f) Comparison of the NH_3_ formation rate of different samples.

To evaluate the electrocatalytic NRR performance of NC/Bi SAs/TiN/CC, linear sweep voltammetry (LSV) measurements were first performed in N_2_‐ and Ar‐saturated 0.1 m Na_2_SO_4_ electrolytes under room temperature and atmospheric pressure (**Figure** **3**a). The LSV curves display the current density in N_2_‐ saturated 0.1 m Na_2_SO_4_ is larger than that in Ar‐saturated one between −0.5 and −0.9 V (versus RHE), indicating that the NRR occurs in a potential range from −0.5 to −0.9 V (versus RHE). Chronoamperometry tests were then performed under the potentials ranging from −0.5 to −0.9 V versus RHE for 2 h in N_2_‐saturated 0.10 m Na_2_SO_4_ solution to further assess the NRR performance of the NC/Bi SAs/TiN/CC and seek the optimized potential for the NRR (Figure S11a, Supporting Information). The ultraviolet–visible (UV–Vis) absorption spectra of electrolytes obtained for each potential colored with indophenol blue reagent were measured (Figure S11b, Supporting Information). The electrolysis product of NH_3_ at a given potential was quantitatively determined by the indophenol blue method (the standard curve for ammonia concentration is shown in Figure S12, Supporting Information). Figure 3b and Table S2, Supporting Information, show the calculated NH_3_ yield rate and corresponding FE of NC/Bi SAs/TiN/CC at diverse potentials. It shows that the maximum NH_3_ yield rate of 76.15 µg mg_cat_^−1^ h^−1^ is achieved at −0.8 V (versus RHE) while the highest FE of 24.60% is obtained at −0.5 V (versus RHE), which is superior to most reported non‐noble metal catalysts and almost exceeds all previously reported Bi‐based catalysts under similar condition (Tables S3 and S4, Supporting Information). Meanwhile, the NH_3_ yield rate of NC/Bi SAs/TiN/CC at −0.8 V (versus RHE) is also higher than that of Nafion containing electrode of NC/Bi SAs/TiN/CC (N) with the same Bi loading (Figure S13, Supporting Information), suggesting the advantage of 3D self‐supported monolithic structure for improving the catalytic activity. Obviously, the FE decreases with the applied potentials shifts to more negative values, mainly due to the competition of the HER on catalyst surface. In addition, the byproduct N_2_H_4_ is not detected in the electrolytes after 2 h by the Watt and Chrisp method (the standard curve for N_2_H_4_ concentration is shown in Figures S14 and S15, Supporting Information), indicating the excellent selectivity for NH_3_ production at different potentials. To eliminate potential experimental errors caused by the self‐disintegration of catalyst and ammonia in atmosphere, series control experiments were carried out. As shown in Figure S16, Supporting Information, the negligible enhancing of signals associated with ammonia is observed in electrolysis at −0.8 V versus RHE for 2 h in Ar‐saturated electrolyte and the NC/Bi SAs/TiN/CC electrode under open circuit potential for 2 h in N_2_‐saturated electrolyte in comparison to the freshly prepared electrolyte without ammonia.

Stability, as a crucial parameter of NRR performance, was verified by consecutive cycling tests and long‐term electrocatalytic tests. As shown in Figure 3c and Figure S17, Supporting Information, the NH_3_ yield rate and Faraday efficiency of NC/Bi SAs/TiN/CC only slightly fluctuate during 7 cycling tests at the applied potential of −0.8 V versus RHE. The average NH_3_ generation rate and Faraday efficiency are calculated to be 71.02 ± 2.65 µg mg_cat_^−1^ h^−1^ and 4.32 ± 0.11%, respectively and the loss of NH_3_ yield rate is only about 6.7% in comparison to the best one. After a long‐term NRR test of 10 h at an applied potential of −0.8 V versus RHE, the current density of NC/Bi SAs/TiN/CC is still kept at 94.2% of the initial value, while for NC/Bi SAs/TiN/CC (N), only 75.2% of current density is retained (Figure S18, Supporting Information). These results reveal the superior stability of NC/Bi SAs/TiN/CC with 3D self‐supported integrated structure for NRR. Additionally, the XRD pattern, SEM images, STEM image, and corresponding EDX elemental mappings of the NC/Bi SAs/TiN/CC after stability test show that the crystalline phase, morphology, structure, and composite have no obvious changes (Figures S19, S20, and S21a–e, Supporting Information). Meanwhile, the AC‐HAADF‐STEM image reveals that most of Bi species in NC/Bi SAs/TiN/CC still exist as single atoms and only a few Bi single atoms agglomerate to nanoclusters (Figure S21f, Supporting Information). Furthermore, the chemical valance state of Bi species in NC/Bi SAs/TiN/CC after long‐term NRR test remains unchanged (Figure S22, Supporting Information). These results further confirm its high stability for NRR.

To further highlight the superiority of NC/Bi SAs/TiN/CC, the chronoamperometry test for NC/Bi NPs/TiN/CC and Bi‐MOF/TiN/CC were conducted at different potentials. And the Bi loadings on NC/Bi NPs/TiN/CC and Bi‐MOF/TiN/CC, measured by ICP‐AES, are 0.26 and 3.56 mg cm^−2^, respectively. (Table S1, Supporting Information). As shown in Figure 3d, the calculated NH_3_ yield rate at −0.8 V versus RHE and Faraday efficiency at −0.5 V versus RHE are 2.88 µg mg_cat_^−1^ h^−1^ and 7.92% for NC/Bi NPs/TiN/CC and 1.21 µg mg_cat_^−1^ h^−1^ and 14.90% for Bi‐MOF/TiN/CC, which are inferior to that of NC/Bi SAs/TiN/CC. The ^1^H nuclear magnetic resonance (^1^H NMR) spectra were measured to verify the origin of produced NH_3_. As exhibited in Figure 3e, doublet coupled peaks of ^15^NH_4_^+^ and triplet coupled peaks of ^14^NH_4_^+^ are observed in the ^1^H NMR spectrum when using ^15^N_2_ and ^14^N_2_ as the feed gas for NRR while the peaks attributed to ^15^NH_4_^+^ and ^14^NH_4_^+^ disappears when Ar is bubbled into the electrolyte or the blank electrolyte is used, confirming the NH_3_ is produced from NRR.^[^ ^45^ ^]^

The NRR performance of NC/Bi SAs/CC, NC/TiN/CC, and TiN/CC were also tested at −0.8 V versus RHE in 0.1 m Na_2_SO_4_ electrolyte to further reveal the actives sites. As shown in Figure 3f, NC/Bi SAs/TiN/CC exhibits the highest NH_3_ yield rate of 76.15 µg mg_cat_^−1^ h^−1^, much higher than those of NC/Bi SAs/CC (28.03 µg mg_cat_^−1^ h^−1^), NC/TiN/CC (12.29 µg mg_cat_^−1^ h^−1^) and bare TiN/CC (0.03 µg mg_cat_^−1^ h^−1^), implying that the Bi SAs supported on TiN should be the main active sites for NRR. Subsequently, the charge transfer resistances (*R*_ct_) of NC/Bi SAs/TiN/CC, NC/Bi NPs/TiN/CC, NC/Bi SAs/CC, NC/TiN/CC, and TiN/CC were obtained by the electrochemical impedance spectroscopy (EIS) test (Figure S23, Supporting Information). Among these catalysts, the NC/Bi SAs/TiN/CC electrode displays the lowest *R*_ct_ of 41.36 Ω, illustrating more efficient NRR kinetics (Table S5, Supporting Information). The low *R*_ct_ of NC/Bi SAs/TiN/CC electrode can be linked with the synergistic effect of NC, Bi SAs, and TiN, which improves the electron transfer property. In addition, the NC/Bi SAs/TiN/CC electrode possesses a larger ECSA of 55.0 mF cm^−2^ as compared to NC/TiN/CC (53.2 mF cm^−2^), TiN/CC (42.8 mF cm^−2^), and carbon cloth (2.1 mF cm^−2^) (Figure S24, Supporting Information), demonstrating more active sites.

In order to verify the electrocatalytic activity and selectivity of different catalysts toward NRR, we further carried out the DFT calculations of the electronic properties and the different mechanisms of NRR on several catalysts. Based on our experimental characterization results, we constructed three catalyst models of Bi (110), BiN_4_‐@C, and Bi_1_/TiN (Figure S25, Supporting Information), in which the Bi (110) represents the Bi nanoparticles in the NC/Bi NPs/TiN/CC catalyst while the BiN_4_‐@C and Bi_1_/TiN respectively refers to the single Bi atom that anchored in the carbon layer and that loaded on the TiN (200) surface in the NC/Bi SAs/TiN/CC catalyst. After optimizing the three models, the binding energies of Bi atoms in BiN_4_‐@C and Bi_1_/TiN were calculated. The calculation results show that the Bi atom supported on the TiN (200) is much more stable than that anchored in the carbon layer (−4.97 eV versus −3.16 eV, Figure S25, Supporting Information). Meanwhile, the binding energy of Bi atom in Bi crystal is close to that anchored in the carbon layer (−3.21 eV versus −3.16 eV). Both results indicate that almost all the Bi atoms can be dispersed as single atom in the catalyst of NC/Bi SAs/TiN/CC, they are probably loaded on the TiN surface or anchored in the carbon layer, which is well in agreement with our characterization results of STEM (Figure 1d,g).

**Figure 4:**
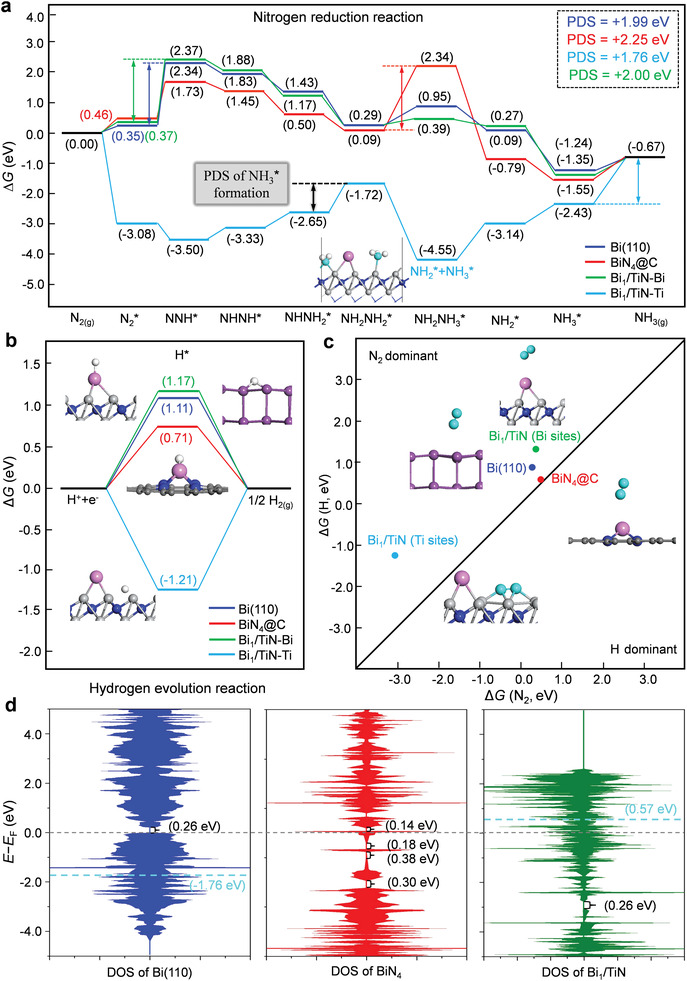
The free‐energy diagrams of NRR (a) and HER (b) on the four different catalyst active sites of Bi (110), BiN_4_@C, Bi_1_/TiN‐Bi, and Bi_1_/TiN‐Ti at the electrode potential of 0 V, in which the PDS of NRR on these four sites are inserted with color fonts; c) the comparison of the adsorption energies of N_2_ molecular and H atom on the four active sites; d) the total electron density of states (DOS) for the three models of Bi (110), BiN_4_@C, and Bi_1_/TiN, in which the band gap is marked by the black font, the P‐band center is inserted with cyan dotted line and font, while the Fermi level is set as zero with grey dotted line. For the inserted structures, the Bi, Ti, N, C, and H atoms are shown in purple, light gray, dark blue, grey, and white. To make a distinction, the single Bi atom in BiN_4_@C and Bi_1_/TiN is shown in pink, while the N atom of N_2_ is shown in cyan.

Generally, there are two different mechanisms for electrocatalytic NRR, that is, the distal pathway and alternating pathway, so the corresponding Gibbs free energies (Δ*G*) for the reaction intermediates of these two mechanisms are further calculated. In view of the Bi_1_/TiN model has two different active sites of Ti and Bi (Bi_1_/TiN‐Ti and Bi_1_/TiN‐Bi), we investigate the two NRR mechanisms on the four different catalyst active sites of Bi (110), BiN_4_‐@C, Bi_1_/TiN‐Ti and Bi_1_/TiN‐Bi (Figures S26 and S27, Supporting Information), and the most stable structures of all the corresponding intermediates on the four different sites are shown in Figures S28–S31, Supporting Information, respectively. According to the calculation results, it is clearly seen that the alternating pathway is the favorable NRR mechanism for all four active sites. Thus, all the alternating pathways are exhibited in **Figure** **4**a for comparison. As shown in Figure 4a, the N_2_ adsorption on Bi_1_/TiN‐Ti is much stronger than that on Bi (110), BiN_4_@C and Bi_1_/TiN‐Bi (−3.08 eV versus 0.35, 0.46, and 0.37 eV), suggesting the activation of N_2_^*^ on the site of Bi_1_/TiN‐Ti is expected to be easier than that of other three active sites. The subsequently detailed studies reveal that the first hydrogenation step of N_2_^*^ + H^+^ + e^−^ → NNH^*^ is the potential‐determining step (PDS) of NRR on the Bi (110) and Bi_1_/TiN‐Bi (1.99 and 2.00 eV), the hydrogenation of NH_2_NH_2_* into NH_2_NH_3_* is the PDS of NRR on the BiN_4_@C (2.25 eV), while the desorption of NH_3_* is the PDS of NRR on the Bi_1_/TiN‐Ti (1.76 eV). Clearly, although the Ti site of Bi_1_/TiN has the lowest PDS of NRR, the so higher PDS value of 1.76 eV results in a low NRR activity. Surprisingly, it is found that the N‐N bond can be cracked easily in the hydrogenation step of NH_2_NH_2_^*^ on the Bi_1_/TiN‐Ti with a lower PDS of 0.93 eV and forms into the surface species of NH_2_^*^ and NH_3_^*^, that is, NH_2_NH_2_^*^ + H^+^ + e^−^ → NH_2_^*^ + NH_3_^*^. In addition, it is also seen that the desorption energy of NH_3_^*^ is very low on the Bi_1_/TiN‐Bi (0.68 eV). Therefore, for the NRR mechanism on the NC/Bi SAs/TiN/CC catalyst, it is inferred that the N_2_ molecule can be promptly hydrogenated into the surface NH_3*_ on the Ti sites of Bi_1_/TiN‐Ti with the potential of 0.93 V, then the surface NH_3_* will be desorbed on the Bi site of Bi_1_/TiN‐Bi with the potential of 0.68 V, and the NH_3_ molecule is further released via the channel between the carbon layer and Bi_1_/TiN; in turn, the agglomeration and loss of Bi atoms can be suppressed by the carbon layer. In a word, the calculation results demonstrate that the synergistic effect between single Bi atom and TiN surface and the protective effect of carbon layer in the catalyst of NC/Bi SAs/TiN/CC enables the excellent activity and stability of NRR, which is consistent with our experiment results.

As is well‐known, the NRR suffers from poor selectivity and lower FE% because of the competition of HER. To further get insight into the good selectivity of NC/Bi SAs/TiN/CC toward NRR, the HER mechanism was also examined on the four different active sites of Bi (110), BiN_4_@C, Bi_1_/TiN‐Bi, and Bi_1_/TiN‐Ti (Figure 4b, the corresponding structures are displayed in Figure S32, Supporting Information), and the comparison of Δ*G*_N2*_ and Δ*G*_H*_ was carried out as well (Figure 4c). Obviously, all these four active sites are in the N_2_ dominant region (Δ*G*_N2*_ < Δ*G*_H*_ at *U* = 0 V), especial for the Bi_1_/TiN‐Ti, which suggests the NRR is more favorable to occur on the Ti site of Bi_1_/TiN rather than HER (1.21 eV versus 0.93 eV).

Furthermore, to deeply understand the excellent catalytic performance of NC/Bi SAs/TiN/CC, the electron properties were further analyzed, including the electron density of states (DOS) of the Bi (110), BiN_4_@C, and Bi_1_/TiN (Figure 4d), as well as the Bader charge of all the different elements in the BiN_4_@C and Bi_1_/TiN (Table S6, Supporting Information). As shown in Figure 4d, the Bi (110) has a band gap of 0.26 eV above the Fermi level and a very low p‐band center of −1.76 eV, however, the Bi_1_/TiN has the same band gap of 0.26 eV below the Fermi level and a very high p‐band center of 0.57 eV, implying that the electrical conductivity of the Bi_1_/TiN is much better than that of the Bi (110), which is well in agreement with the experiment results. For the BiN_4_@C, it can be clearly seen that there are three (<0.38 eV) and one (0.14 eV) band gaps below and above the Fermi level, respectively, suggesting the doping of Bi atom can split the p‐band of the carbon layer and reduce its electrical conductivity. In the BiN_4_@C and Bi_1_/TiN, the charge of the single Bi atom is calculated to be +1.50 and −0.68, respectively (Table S6, Supporting Information), manifesting that the electrons transfer from the Bi atom to the N atoms in the former while the electrons transfer from the TiN substrate to the Bi atom in the latter. Totally, the mechanism calculations of NRR and HER on the four different active sites, as well as the electron property analysis of the catalyst models can well explain the experiment results, the synergistic effect of the TiN substrate, the single Bi atom, and the carbon layer can enable the excellent activity and selectivity of NRR on the catalyst of NC/Bi SAs/TiN/CC.

## 3 Conclusion

In summary, single Bi atoms incorporated titanium nitride nanorods encapsulated in nitrogen‐doped carbon layer supported on a carbon cloth (NC/Bi SAs/TiN/CC) have been successfully synthesized as an efficient self‐supported electrode for electrocatalytic NRR. The optimized NC/Bi SAs/TiN/CC electrode exhibits a promising ammonia yield of 76.15 µg mg_cat_^−1^ h^−1^ and a high FE of 24.60% in 0.1 m Na_2_SO_4_, which is several times better than NC/Bi NPs/TiN/CC and NC/Bi SAs/CC and higher than the overwhelming majority of the reported Bi‐based catalyst. The superior electrocatalytic NRR performance originates from atomically dispersed active sites, high mass/charge transfer efficiency, outstanding conductivity, as well as the strong synergistic effect between Bi SAs and TiN substrate, which could simultaneously promote the hydrogenation of N_2_ molecule into NH_3_^*^ on the TiN substrate and the desorption of NH_3_^*^ from single‐atomic Bi sites and thus boosts ENRR. The design demonstrated herein opens up a feasible pathway to fabricate various 3D self‐supported single‐atomic electrocatalysts by rationally engineering their structure, composition, and electronic configuration of single atoms for various electrochemical reactions.

## Conflict of Interest

The authors declare no conflict of interest.

## Supporting information

Supporting Information

## Data Availability

Research data are not shared.
