# Supplementary material for: Boosting Nitrogen Reduction Reaction via Electronic Coupling of Atomically Dispersed Bismuth with Titanium Nitride Nanorods
Source: Adv Sci (Weinh). 2021 Dec 2;9(4):2104245. doi: 10.1002/advs.202104245 (PMC8811825; doi:10.1002/advs.202104245)
Supplement: Supplementary file 1 — Supporting Information [file ADVS-9-2104245-s001.pdf]

WILEY-VCH

ADVANCED SCIENCE
Open Access

# Supporting Information

for *Adv. Sci.*, DOI: 10.1002/advs.202104245

## Boosting Nitrogen Reduction Reaction via Electronic Coupling of Atomically Dispersed Bismuth with Titanium Nitride Nanorods

*Zichao Xi, Ke Shi, Xuan Xu, Peng Jing, Baocang Liu,* Rui Gao,* and Jun Zhang**

Copyright WILEY-VCH Verlag GmbH & Co. KGaA, 69469 Weinheim, Germany, 2018.

# Supporting Information

## Boosting Nitrogen Reduction Reaction via Electronic Coupling of Atomically Dispersed Bismuth with Titanium Nitride Nanorods

*Zichao Xi,*[ǂ] *Ke Shi,*[ǂ] *Xuan Xu, Peng Jing, Baocang Liu,*[*] *Rui Gao,*[*] *and Jun Zhang*[*]

**Table of contents:**

Section 1. Experimental details

Section 2. Supplementary Tables

Section 3. Supplementary Figures

Section 4. Supplementary References

## Section 1. Experimental details

### Materials

Methanol ($CH_3OH$, ≥ 99.5%), ethanol ($C_2H_5OH$, ≥ 99.7%) and acetone ($CH_3COCH_3$, ≥ 99.5%) were purchased from T·JKEMAO Chemical Reagents Co., Ltd. N, N-dimethylformamide (DMF, 99.9%), salicylic acid ($C_7H_6O_3$, 99%), sodium citrate ($Na_3C_6H_5O_7·2H_2O$, 99%), sodium hypochlorite (NaClO, active chlorine >10%), sodium nitroferricyanide dehydrate ($C_5FeN_6Na_2O·2H_2O$, 99%), sodium sulfate anhydrous ($Na_2SO_4$, ≥ 99%), potassium iodide (KI, 99 %), bismuth nitrate pentahydrate

($Bi(NO_3)_2 \cdot 5H_2O$, 99%), and carbon cloth (40×40 cm) were obtained from Innochem Science & Technology Co., Ltd. Sodium hydroxide (NaOH, ≥ 98%) and 1,3,5-benzenetricarboxylic acid ($C_6H_3(CO_2H)_3$, 98%) were received from Shanghai Aladdin Biochemical Technology Co., Ltd. Aniline (99+%), p-benzoquinone ($C_6H_4O_2$. 98+%), and dicyandiamide ($C_2H_4N_4$, 99%) were bought from Alfa Aesar (China). P-dimethylaminobenzaldehyde ($C_9H_{11}NO$, 99+%) was purchased from Acros Organics. Nafion 211 was obtained from Gaoss Union Co., Ltd. Titanium n-butoxide ($C_{16}H_{36}O_4Ti$, ≥ 98%) and hydrochloric acid (HCl, 37wt%) were received from Sinopharm Chemical Reagent Co., Ltd. Ar (99.999%) gas and $^{14}N_2$ (99.999%) gas were bought from Beijing Oxygen Plant specialty gases institute Co., Ltd. $^{15}N_2$ (99 atom% $^{15}N$) gas was obtained from Wuhan Newradar special gas Co., Ltd. All reagents were used as received without further purification. Aldrich. All reagents were used without further purification. The water used in the experiments was purified by the Millipore system.

**Characterizations**

X-ray diffraction (XRD) was performed on a PANalytical Empyrean diffractometer with Cu Kα radiation (λ = 1.5405 Å). Scanning electron microscopy (SEM) images were recorded on a Hitachi S-4800 field emission scanning electron microscope. Transmission electron microscopy (TEM) and Aberration-corrected high angle annular dark-field scanning TEM (AC-HAADF-STEM) characterizations were carried out on a JEOL ARM200F field-emission transmission electron microscope equipped with an EDX detector at an acceleration voltage of 200 kV. X-ray photoelectron spectroscopy (XPS) measurements were conducted on a Thermo

Scientific ESCALAB $Xi^+$ spectrometer equipped with two ultrahigh vacuum (UHV) chambers. All spectra were calibrated with the C=C peak of C 1s orbitals as 284.6 eV. Ultraviolet-visible (UV-Vis) spectroscopy measurements were performed on a HITACHI U-3900 spectrometer. $^1H$ nuclear magnetic resonance (NMR) spectra were obtained using a Bruker Advancell 500 MHz system. Raman data were collected by. Raman spectra were captured on a Thermo Fisher DXR spectrometer with a 532 nm excitation laser. Inductively coupled plasma Optical Emission Spectrometer (ICP-OES) analysis was conducted by an Agilent 720ES.

**Preparation of TiN/CC**

Typically, 0.8685 g of titanium n-butoxide (TBT) was dissolved in absolute ethanol (50 mL). A piece of cleaned CC (3.5 cm × 2 cm) was immersed into the above solution and ultrasonic treated for 30 min and dried at 400 ºC for another 30 min in a tube furnace. The obtained CC was then transferred into a 50 mL Teflon-lined stainless autoclave filled with the growth solution containing TBT (1.6 mL), HCl (15 mL), and acetone (16 mL). The autoclave was sealed and heated at 200 °C for 80 min in an electric oven. Afterwards, the autoclave was cooled to room temperature; the product was then collected, washed with ethanol three times, and dried at 60 °C for 8 h to obtain $TiO_2$/CC. After further annealing at 900 °C for 2 h in ammonia atmosphere, the TiN/CC was obtained.

**Preparation of BiOI/TiN/CC**

BiOI/TiN/CC was prepared by an electrochemical deposition method. Typically, 6.64 g of KI was dissolved in deionized water (100 mL) and the pH of the solution was

adjusted to 1.7 by concentrated nitric acid, then 1.94 g of $Bi(NO_3)_3·5H_2O$ was added under stirring to obtain solution A. Then, 0.9944 g of p-benzoquinone was dissolved in absolute ethanol (40 mL) to achieve solution B. Afterwards, mixing solution A and solution B and stirring for 20 min to form the precursor solution. The preparation of BiOI/TiN/CC was carried out in an electrochemical cell in the precursor solution at 25 °C. A Pt foil, an Ag/AgCl electrode, and the as-prepared TiN/CC served as the counter, the reference, and the working electrode, respectively. A constant voltage of -1.0 V vs Ag/AgCl was applied for 1000 s to deposit BiOI on TiN/CC to form BiOI/TiN/CC. Finally, the BiOI/TiN/CC was washed with deionized water and drying at 60 °C for 6 h.

**Preparation of PANI/Bi-MOF/TiN/CC**

Bi-MOF/TiN/CC was synthesis by an ion-exchange method. Typically, 2.625 g of trimesic acid ($H_3BTC$) was added into in a mixture solution of DMF (24 mL) and methanol (6 mL) in a 50 mL Teflon autoclave. Next, the BiOI/TiN/CC was put into the mixture solution vertically. Then, the autoclave was sealed and heated at 120 °C for 24 h. After cooling down to room temperature, the Bi-MOF/TiN/CC was taken out, washed with methanol, and dried at 60 °C. Polyaniline film on Bi-MOF/TiN/CC (PANI/Bi-MOF/TiN/CC) was achieved by electrodeposition in a solution containing 2 mL of aniline and 0.05 M of $Na_2SO_4$ at 0.8 mA $cm^{-2}$ for 1.0 h at room temperature.

**Preparation of NC/Bi SAs/TiN/CC and NC/Bi SAs/TiN/CC (N)**

The as-prepared PANI/Bi-MOF/TiN/CC precursor and 2.0 g dicyandiamide (DCDA) in two porcelain boats were placed at two separate positions in the tube

furnace and heated to 900 °C at a ramping rate of 2 °C $min^{-1}$ for 1 h in a stream of $N_2$ to achieve NC/Bi SAs/TiN/CC. The Bi loading on NC/Bi SAs/TiN/CC is measured to be 0.023 mg $cm^{-2}$ by ICP-AES. For comparison, the NC/Bi SAs/TiN/CC (N) electrode was also prepared via a drop coating method. Typically, 10.5 mg of NC/Bi SAs/TiN composite nanorods were scraped down from CC and prepared into ink by using water (0.25 mL) and ethanol (0.25 mL) as solvent and Nafion (25 μL) as binder under ultrasound. After dripping the ink on CC (1 cm × 2 cm) and dried under a baking lamp, the Nafion containing electrode of NC/Bi SAs/TiN/CC (N) with the same Bi loading as the NC/Bi SAs/TiN/CC electrode was obtained.

**Preparation of NC/Bi NPs/TiN/CC**

The as-prepared PANI/Bi-MOF/TiN/CC precursor and 2.0 g dicyandiamide (DCDA) in two porcelain boats were placed at two separate positions in the tube furnace and heated to 700 °C at a ramping rate of 2 °C $min^{-1}$ for 1 h in a stream of $N_2$ to yield NC/Bi NPs/TiN/CC.

**Preparation of NC/Bi SAs/CC**

The as-prepared PANI/Bi-MOF/CC precursor and 2.0 g dicyandiamide (DCDA) in two porcelain boats were placed at two separate positions in the tube furnace and heated to 900 °C at a ramping rate of 2 °C $min^{-1}$ for 1 h in a stream of $N_2$ to yield NC/Bi SAs/CC.

**Preparation of NC/TiN/CC**

The as-prepared PANI/TiN/CC precursor and 2.0 g dicyandiamide (DCDA) in two porcelain boats were placed at two separate positions in the tube furnace and heated to

900 °C at a ramping rate of 2 °C $min^{-1}$ for 1 h in a stream of $N_2$ to yield NC/TiN/CC.

**Preparation of NRR measurement**

All NRR measurements were carried out in an H-type cell using a three-electrode system by an electrochemical workstation (CHI 760E). An Ag/AgCl electrode, a graphite rod, the as-prepared NC/Bi SAs/TiN/CC (1.2 cm × 0.6 cm) served as as the reference electrode, the counter electrode, the working electrode, respectively. In this work, we converted all the applied potentials into the reference hydrogen electrode (RHE) using the formulation (E (vs. RHE) = E (vs. Ag/AgCl) + 0.197 V + 0.0591 V × pH).

**NRR measurements**

All NRR measurements were evaluated in a gas-tight standard three-electrode H-type cell which concludes 40mL of 0.1 M $Na_2SO_4$ electrolyte. And the cathodic and anodic compartments were separated by a Nafion 211 membrane (25.4 μm), which has been treated with 5 w% $H_2O_2$, 0.5 M $H_2SO_4$, and deionized water at 80 °C for 1 h before using. Before electrolysis, cyclic voltammetry scanning was performed at a rate of 50 mV $s^{-1}$ for 20 cycles from -0.8 V to -1.5 V to stabilize the catalyst and remove the potential impurities in the working electrode. Meanwhile, ultrahigh purity $^{14}N_2$ (99.999%) or $^{15}N_2$ (99.999%) was continuously bubbled into the electrolyte for 30 min to ensure the removal of residual air in the reaction system. For NRR experiments, potentiostatic tests were conducted at different potentials ranged from -0.5 V to -0.9 V vs. RHE for 2 h in $N_2$ saturated 0.1 M $Na_2SO_4$ solution. During NRR, high-purity $N_2$ was continuously fed into the cathodic compartment. For comparison, potentiostatic

tests were also carried out in Ar-saturated (99.999 %) in 0.1 M $Na_2SO_4$ electrolyte solution. All tests were performed at room temperature (25°C) under magnetic stirring.

**Calibration curve of $NH_3$ in 0.1 M $Na_2SO_4$ solution**

First, 0.3146 g of $NH_4Cl$ (pretreat at 105 °C for 4h) was immersed in 0.1 M $Na_2SO_4$ solution (100 mL) to obtain a 1000 $\mu g_{NH3}$/mL standard solution. Then, 1 mL of 1000 $\mu g_{NH3}$/mL standard solution was added into a 100 mL volumetric flask and added 0.1 M $Na_2SO_4$ solution to the scale mark. Then, 0.25, 0.5, 1.0, 1.5, and 2.0 mL of the above solution were added into 25 mL volumetric flasks and diluted with 0.1 M $Na_2SO_4$ solution to the scale mark. Thus, 0.1, 0.2, 0.4, 0.6, and 0.8 $\mu g_{NH3}$ $mL^{-1}$ standard solutions were obtained.

**Ammonia detection**

**(a) Indophenol blue reagent**

Solution A (Chromogenic reagent): 5 wt% of salicylic acid and 5 wt% of sodium citrate were dissolved in 1 M NaOH solution.

Solution B (Oxidation reagent): 3.5 mL of sodium hypochlorite (available chlorine concentration 10-15 %) was added in 100 mL of deionized water.

Solution C (Catalytic reagent): 0.2 g of $C_5FeN_6Na_2O$ was dissolved in 20 mL of deionized water.

**(b) Quantification of $NH_3$**

2 mL of test solutions was taken out and transferred into a 25 mL conical flask. Then, 2 mL of solution A, 1 mL of solution B, and 0.2 mL of solution C were added in sequence. After being incubated with indophenol blue indicator at 25 °C for 1 h, the

absorbance at 655 nm for each solution was collected with a UV-Vis spectrophotometer. The concentrations of $NH_3$ in the test solutions were calculated directly from the calibration curve.

**Quantification of hydrazine**

Watt and Chrisp method were employed to quantify the possiblely existed hydrazine. 5.99 g of para-(dimethylamino) benzaldehyde was dissolved in a mixture of concentrated HCl (30 mL) and ethanol (300 mL) to obtain the chromogenic reagent. After adding the chromogenic reagent in the test electrolytes and incubating for 10 min, the absorbance at 455 nm for each electrolyte was measured with a UV-Vis spectrophotometer. The concentrations of hydrazine in the test electrolytes were determined from the calibration curve.

**$^{15}$N isotopic labeling Experiment**

$^{15}N_2$ (Wuhan Newradar special gas Co., Ltd. ≥ 99.9 atom% $^{15}N_2$) gas was employed as the feed gas to clarify the source of ammonia. After $^{15}N_2$ electroreduction for 3.5 h at -0.8 V (vs. RHE) in 0.1 M $Na_2SO_4$ solution, 0.1 M HCl solution was added into the obtained $^{15}NH_4^+$ electrolyte solution to adjust the pH value to 2.0 and d6-DMSO was added to achieve sufficient lock signal. The above treated $^{15}NH_4^+$ electrolyte solution was used for $^{1}$H NMR measurement.

**Calculation of $NH_3$ yield rate and Faradaic efficiency**

The average yield rate of $NH_3$ was calculated using the following formula:

$$r(NH_3) = \frac{c(NH_3) \times V}{t \times m}$$

Where c is the measured $NH_3$ concentration, V is the volume of electrolyte, t is the

time for NRR, and m is the loading of active species on CC (without including TiN).

The geometric average yield rate of $NH_3$ was calculated using the following equation:

$$r(NH_3) = \frac{c(NH_3) \times V}{t \times A}$$

Where A is the geometric area of the working electrode.

The Faradaic efficiency of NRR was calculated using the following formula:

$$\text{Faradaic efficient} = \frac{3 \times F \times c(NH_3) \times V}{17 \times Q}$$

Where 3 is the electron number of forming an ammonia molecule, F is the Faraday constant (96485 $C \cdot mol^{-1}$), c is the measured $NH_3$ concentration, V is the volume of electrolyte, Q is the total charge used for the electrodes.

## Computational Details

*DFT Methods:*

In this computation work, all spin-polarized first-principles calculations were performed using the Vienna Ab Initio simulation package (VASP).[S1, S2] The electron-ion interaction was described by the projector augmented wave (PAW) method,[S3, S4] and the electron exchange and correlation energies were treated within the generalized gradient approximation in the Perdew-Burke-Ernzerhof formalism (GGA-PBE).[S5] All the models were constructed with a vacuum slab of 15 Å in z-direction. The energy cutoff of plane wave basis was set as 450 eV. Besides, we utilized a Monkhorst-Pack mesh of 3×3×1 for the calculations, and all of the calculations were converged to the energy change of $1 \times 10^{-5}$ eV. The Van der Waals interaction ($D_3$) was considered in this work due to the weak adsorption of $N_2$ molecules on the Bi atoms of catalyst surface.

As following 14 elementary steps from (1) to (14) (* in sign of the adsorption site), the whole catalytic cycle of the alternating and distal pathway of NRR were composed, and the changes of Gibbs free energy (Δ*G*) of all these steps were calculated.

The alternating pathway:

$N_2(g) + * \rightarrow N_2^*$ (1)

$N_2^* + H+ + e– \rightarrow NNH^*$ (2)

$NNH^* + H^+ + e^- \rightarrow NHNH^*$ (3)

$NHNH^* + H^+ + e^- \rightarrow NHNH_2^*$ (4)

$NHNH_2^* + H^+ + e^- \rightarrow NH_2NH_2^*$ (5)

$NH_2NH_2^* + H^+ + e^- \rightarrow NH_2NH_3^*$ (6)

$NH_2NH_3^* \rightarrow NH_2^* + NH_3(g)$ (7)

$NH_2^* + H^+ + e^- \rightarrow NH_3^*$ (8)

$NH_3^* \rightarrow * + NH_3(g)$ (9)

The distal pathway:

$N_{2(g)} + * \rightarrow N_2^*$ (1)

$N_2^* + H^+ + e^- \rightarrow NNH^*$ (2)

$NNH^* + H^+ + e^- \rightarrow NNH_2^*$ (10)

$NNH_2^* + H^+ + e^- \rightarrow NNH_3^*$ (11)

$NNH_3^* \rightarrow N^* + NH_3(g)$ (12)

$N^* + H^+ + e^- \rightarrow NH^*$ (13)

$NH^* + H^+ + e^- \rightarrow NH_2^*$ (14)

$NH_2^* + H^+ + e^- \rightarrow NH_3^*$ (8)

$NH_3^* \rightarrow * + NH_3(g)$ (9)

The free energy of a species was calculated according to $\Delta G = \Delta E + \Delta E_{ZPE} - T\Delta S$, where $\Delta E$ is the total reaction energy change calculated using DFT calculations, $\Delta E_{ZPE}$ is the correction in zero-point energy, T is the temperature (298.15 K), and the $\Delta S$ is the change in entropy, which values is calculated for the absorbed species using harmonic vibrational frequency analysis. The gas phase values of entropy for $N_2$ and $NH_3$ molecule gas are taken from standard thermodynamic tables, and their ZPE correction is 0.59 and 0.60 eV. What's more, the free energy of one $H^+$ was calculated by $G_{H^+} = 1/2G_{H2}$.

*DFT Models:* In **Figure S22**, the calculated lattice constant of pure Bi cell is a = b = c =4.731 Å, α = β = γ = 57.4446 °; the lattice constant of the TiN cell is a = b = c = 3.00412 Å and α = β = γ= 60 °; and the top and side view of three catalyst models of Bi (110), $BiN_4$@C and $Bi_1$/TiN were shown. Totally, the Bi (110) has 32 Bi atoms, in which 16 Bi were fixed; the $BiN_4$@C has 66 C atoms, 4 N atoms and single Bi atoms, in which all atoms were relaxed; the $Bi_1$/TiN has 64 Ti atoms, 64 N atoms and single Bi atom, in which 32 Ti and 32 N atoms were fixed.

**Section 2. Supplementary Tables**

**Table S1.** The Bi loadings on different electrodes measured by ICP-AES.

| Electrode | NC/Bi SAs/TiN/CC | NC/Bi NPs/TiN/CC | Bi-MOF/TiN/CC |
|---|---|---|---|
| Bi loading (mg $cm^{-2}$) | 0.023 | 0.26 | 3.56 |

**Table S2.** NRR performance of the NC/Bi SAs/TiN/CC.

| Potential (V vs. RHE) | -0.5 | -0.6 | -0.7 | -0.8 | -0.9 |
|---|---|---|---|---|---|
| $NH_3$ yield rate ($\mu g\ mg_{cat}^{-1}h^{-1}$) | 30.75 | 41.40 | 45.41 | 76.15 | 42.88 |
| Faradaic efficiency (%) | 24.6 | 17.49 | 5.42 | 2.94 | 0.40 |

**Table S3.** Comparison of NRR performances of NC/Bi SAs/TiN/CC with reported NRR catalysts under ambient conditions.

| Electrocatalyst | Electrolyte | $NH_3$ yield rate | FE (%) | Reference |
|---|---|---|---|---|
| NC/Bi SAs/TiN/CC | 0.1 M $Na_2SO_4$ | 23.91 μg $cm^{-2}$ $h^{-1}$<br>75.16 μg $mg_{cat.}^{-1}$ $h^{-1}$ | 24.6 | Our work |
| NC-Cu SA | 0.1 M KOH | 53.3 μg $mg_{cat.}^{-1}$ $h^{-1}$ | 13.8 | [S6] |
| | 0.1 M HCl | 49.3 μg $mg_{cat.}^{-1}$ $h^{-1}$ | 11.7 | |
| $Y_1$/NC | 0.1 M HCl | 23.2 μg $cm^{-2}$ $h^{-1}$ | 12.1 | [S7] |
| $Sc_1$/NC | | 20.4 μg $cm^{-2}$ $h^{-1}$ | 11.2 | |
| Fe-SAs/LCC | 0.1 M KOH | 32.1 μg $mg_{cat.}^{-1}$ $h^{-1}$ | 29.3 | [S8] |
| SA-Mo/NPC | 0.1 M KOH | 34.0 μg $mg_{cat.}^{-1}$ $h^{-1}$ | 14.6 | [S9] |
| $Fe_1$-N-C | 0.1 M HCl | $1.56\times10^{-11}$ mol $cm^{-2}$ $s^{-1}$ | 4.51 | [S10] |
| FePc/C | 0.1 M $Na_2SO_4$ | 10.25 μg $mg_{cat.}^{-1}$ $h^{-1}$ | 14.17 | [S11] |
| Pt SAs/$WO_3$ | 0.1 M $K_2SO_4$ | 342.4 μg $mg_{Pt}^{-1}$ $h^{-1}$ | 31.1 | [S12] |
| MoSAs-$Mo_2C$/NCNTs | 0.005 M $H_2SO_4$ + 0.1 M $K_2SO_4$ | 16.1 μg $cm^{-2}$ $h^{-1}$ | 7.1 | [S13] |
| S-NV-$C_3N_4$ | 0.5 M $LiClO_4$ | 32.7 μg $mg_{cat.}^{-1}$ $h^{-1}$ | 14.1 | [S14] |
| N-doped porous carbon | 0.005 M $H_2SO_4$ | 1.31 mmol $h^{-1}$ $g^{-1}$ | 9.98 | [S15] |
| MOF derived N-doped carbon | 0.1 M KOH | $3.4\times10^{-6}$ mol $cm^{-2}$ $h^{-1}$ | 10.2 | [S16] |
| 2D boron nanosheets | 0.1 M $Na_2SO_4$ | 13.22 μg $mg_{cat.}^{-1}$ $h^{-1}$ | 4.04 | [S17] |
| Red phosphorus nanoribbons | 0.1 M $Na_2SO_4$ | 15.4 μg $mg_{cat.}^{-1}$ $h^{-1}$ | 9.4 | [S18] |
| FeS@$MoS_2$/CFC | 0.1 M HCl | 8.45 μg $cm^{-2}$ $h^{-1}$ | 2.96 | [S19] |
| $MoS_2$/$C_3N_4$ | 0.1 M $Na_2SO_4$ | 19.86 μg $mg_{cat.}^{-1}$ $h^{-1}$ | 6.87 | [S20] |
| 2D layered NV-$W_2N_3$ | 0.1 M KOH | 11.66 μg $mg_{cat.}^{-1}$ $h^{-1}$ | 11.67 | [S21] |
| $Bi_2Te_3$ nanoplates | 0.1 M KOH | 3.9 μg $cm^{-2}$ $h^{-1}$ | 4.3 | [S22] |
| Cu-$TiO_2$ | 0.5 M $LiClO_4$ | 21.31 μg $mg_{cat.}^{-1}$ $h^{-1}$ | 21.99 | [S23] |
| Hydroxyl-rich $Ti_3C_2T_X$ QDs | 0.1 M HCl | 62.94 μg $mg_{cat.}^{-1}$ $h^{-1}$ | 13.3 | [S24] |
| np-$PdH_{0.43}$ | 0.1 M PBS | 20.4 μg $mg_{cat.}^{-1}$ $h^{-1}$ | 43.6 | [S25] |
| Mo-Co/NC | 0.1 M $Na_2SO_4$ | 89.8 μmol $h^{-1}$ $g_{cat}^{-1}$ | 13.5 | [S26] |
| VP/V foil | 0.1 M HCl | $8.35\times10^{-11}$ mol $cm^{-2}s^{-1}$ | 22 | [S27] |

**Table S4.** Comparison of NRR performances of NC/Bi SAs/TiN/CC with other reported Bi-based NRR catalysts under ambient conditions.

| Electrocatalyst | Electrolyte | $NH_3$ yield rate | FE (%) | Reference |
|---|---|---|---|---|
| NC/Bi SAs/TiN/CC | 0.1 M $Na_2SO_4$ | 23.91 μg $h^{-1}cm^{-2}$ / 75.16 μg $h^{-1}mg_{cat}^{-1}$ | 24.6 | Our work |
| Bi NCs | 0.5 M $K_2SO_4$ | 200 mmol $g^{-1}$ $h^{-1}$ / 0.052 mmol $cm^{-2}$ $h^{-1}$ | 66 | [S28] |
| Bi@C nanosheets | 0.1 M $Na_2SO_4$ | 4.22 ± 0.33 μg $h^{-1}$ $mg^{-1}$ | 15.1 | [S29] |
| B-doped Bi nanoroll | 0.05 M $H_2SO_4$ | 29.2 $mg_{NH3}$ $g_{cat}^{-1}$ $h^{-1}$ | 8.3 | [S30] |
| Au (111) @$Bi_2S_3$ nanorod | 0.1 M $Na_2SO_4$ | 45.57 μg $h^{-1}$ $mg_{cat.}^{-1}$ | 3.1 | [S31] |
| Multi-yolk-shell bismuth@porous carbon | 0.1 M HCl | 28.63μg $mg_{cat}^{-1}$ $h^{-1}$ | 10.58 | [S32] |
| Bi nanodendrites | 0.1 M HCl | 25.86 μg $mg_{cat}^{-1}$ $h^{-1}$ | 10.8 | [S33] |
| $Bi_4V_2O_{11}/CeO_2$ | 0.1 M HCl | 23.21 μg $mg_{cat}^{-1}$ $h^{-1}$ | 10.16 | [S34] |
| Au nanoparticles on Bi nanosheets | 0.1 M HCl | 20.39 μg $mg_{cat}^{-1}$ $h^{-1}$ | 15.53 | [S35] |
| β-$Bi_2O_3$ | 0.1 M $Na_2SO_4$ | 19.92 μg $mg_{cat}^{-1}$ $h^{-1}$ | 4.3 | [S36] |
| 2D mosaic bismuth nanosheets | 0.1 M $Na_2SO_4$ | 2.54 μg $cm^{-2}$ $h^{-1}$ / 13.23 μg $mg_{cat}^{-1}$ $h^{-1}$ | 10.46 | [S37] |
| Bi ultrathin nanosheets | 0.1 M $Na_2SO_4$ | 11.11 μg $mg_{cat}^{-1}$ $h^{-1}$ | 14.14 | [S38] |
| Abundant edge sites bismuth nanosheets | 0.1 M $NaHCO_3$ | 12.49 μg $mg_{cat}^{-1}$ $h^{-1}$ | 7.09 | [S39] |
| Bi-$CeO_2$/CPs | 0.5 M $K_2SO_4$ | 6.29 μg $cm^{-2}$ $h^{-1}$ | 8.56 | [S40] |
| In suit Fragmented bismuth nanoparticles | 0.1 M $Na_2SO_4$ | 3.25 μg $cm^{-2}$ $h^{-1}$ | 12.11 | [S41] |
| OVs $BiVO_4$ | 0.2 M $Na_2SO_4$ | 8.60 μg $mg_{cat}^{-1}$ $h^{-1}$ | 10.04 | [S42] |
| Plasma R-O-Bi | 0.2 M $Na_2SO_4$ | 5.453 μg $mg_{Bi}^{-1}$ $h^{-1}$ | 11.68 | [S43] |
| $Bi_2O_3$/FEG | 0.1 M $Na_2SO_4$ | 4.21 μg $cm^{-2}$ $h^{-1}$ | 11.2 | [S44] |
| Bi nanosheet array | 0.1 M HCl | 6.89×$10^{-11}$ mol $cm^{-2}$ $s^{-1}$ | 10.26 | [S45] |

**Table S5.** Comparison of the $R_s$ and $R_{ct}$ of different electrodes in 0.1 M $Na_2SO_4$

solution.

| **Electrocatalyst** | **$R_s$ (Ω)** | **$R_{ct}$ (Ω)** |
|---|---|---|
| NC/Bi SAs/TiN/CC | 31.20 | 41.36 |
| NC/Bi NPs/TiN/CC | 30.92 | 85.89 |
| NC/Bi SAs/CC | 35.32 | 290 |
| NC/TiN/CC | 32.77 | 91.32 |
| TiN/CC | 34.71 | 229 |

**Table S6.** The average inverse Bader charge of all the elements in graphite-$BiN_4$ and $Bi_1$/TiN models.

| Models | inverse Bader charge (\|e\|) | | | |
|---|---|---|---|---|
| | Bi | Ti | N | C |
| $BiN_4$@C | +1.50 | / | −1.21 | +0.05 |
| $Bi_1$/TiN | −0.68 | +3.47 | −3.45 | / |
| TiN(200) | / | +3.46 | −3.46 | / |

**Note:** To avoid misunderstanding, the inverse Bader charge is given here, which can be seen as the charge of the different atoms. In other words, the positive value of the inverse Bader charge represents the valence state of the corresponding atom is positive, the electron has been lost from the atom.

**Section 3. Supplementary Figures**

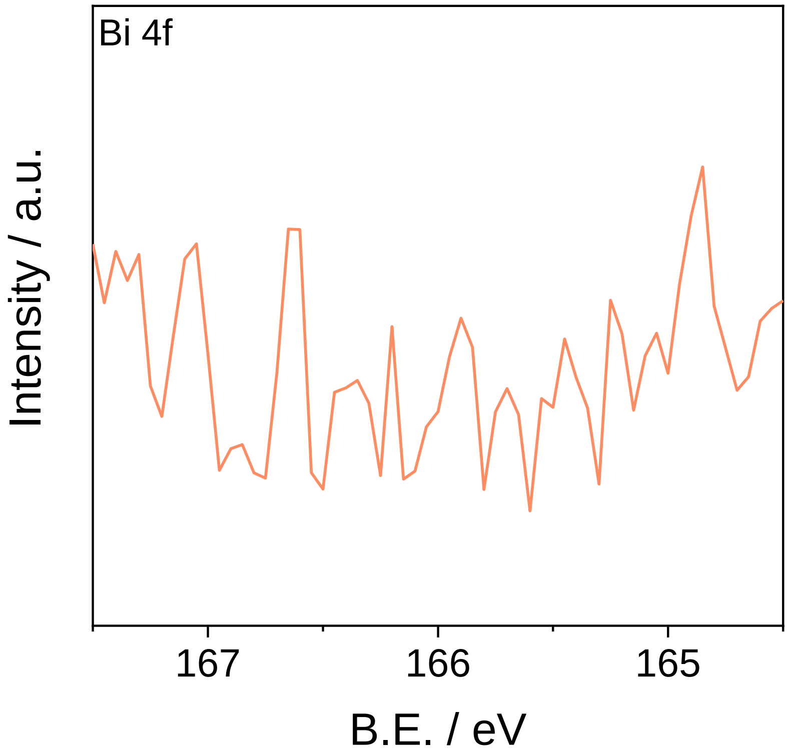


**Figure S1.** XPS spectrum of Bi 4f for the sample fabricated via the similar approach as that of NC/Bi SAs/TiN/CC except for using Bi-MOF/TiN/CC without PANI layer as precursor.

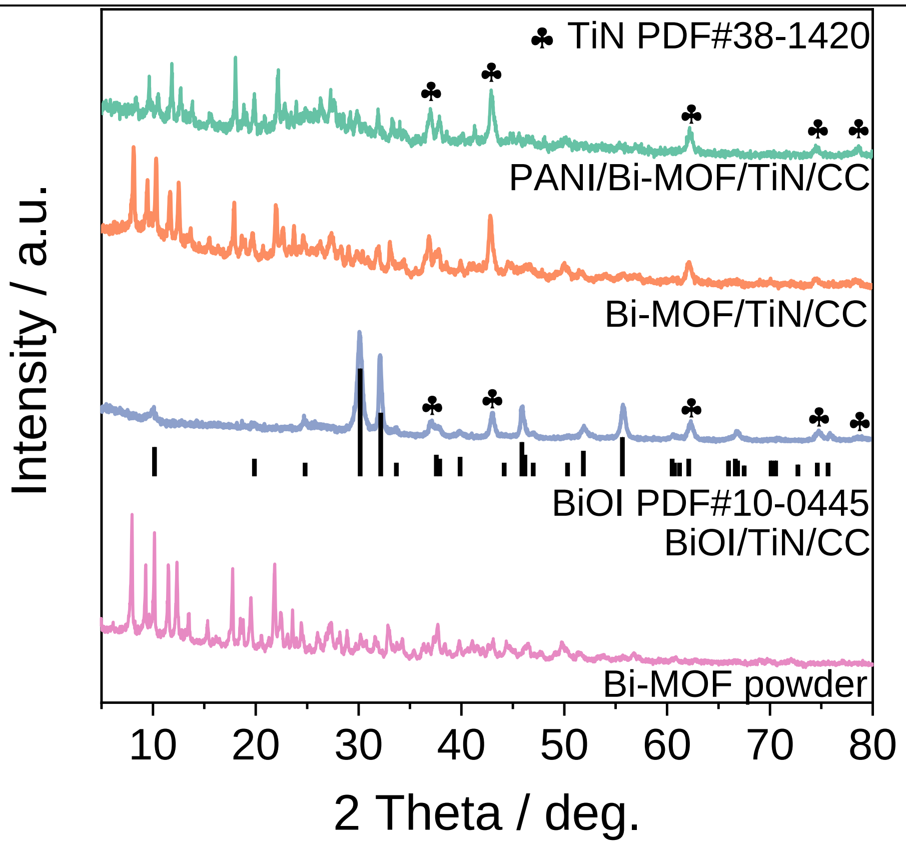


**Figure S2.** XRD patterns of Bi-MOF powder, BiOI/TiN/CC, Bi-MOF/TiN/CC, and PANI/Bi-MOF/TiN/CC.

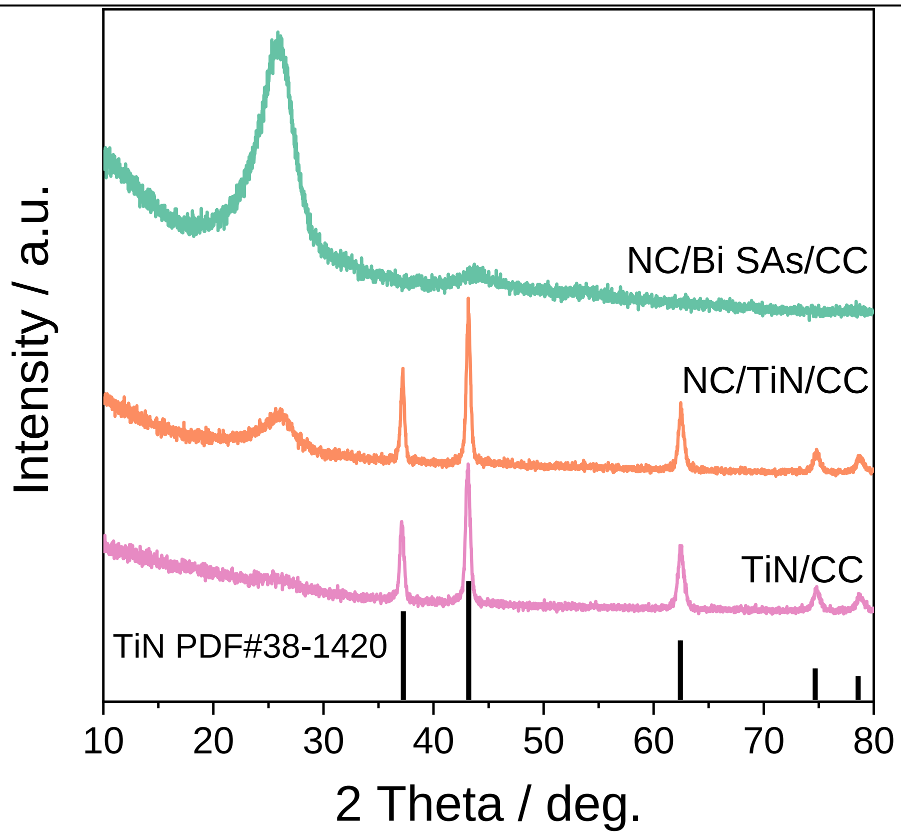


**Figure S3.** XRD patterns of TiN/CC, NC/TiN/CC, and NC/Bi SAs/CC.

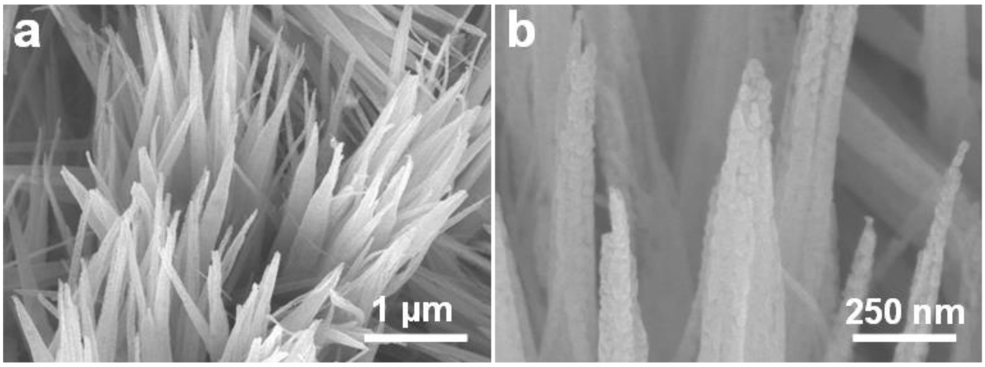


**Figure S4.** (a) Low and (b) high-resolution SEM images of TiN/CC.

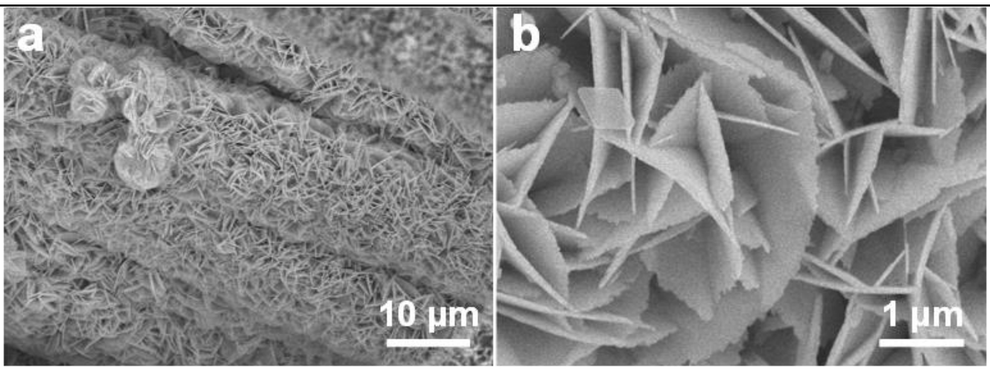


**Figure S5.** (a) Low and (b) high-resolution SEM images of BiOI/TiN/CC.

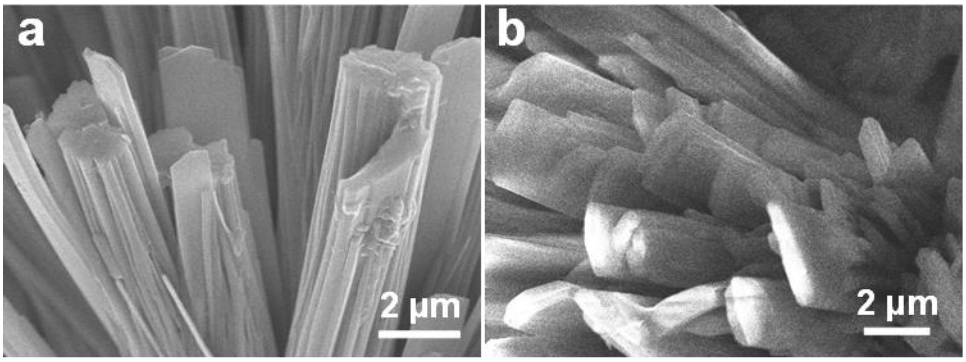


**Figure S6.** SEM images of (a) Bi-MOF/TiN/CC and (b) PANI/Bi-MOF/TiN/CC.

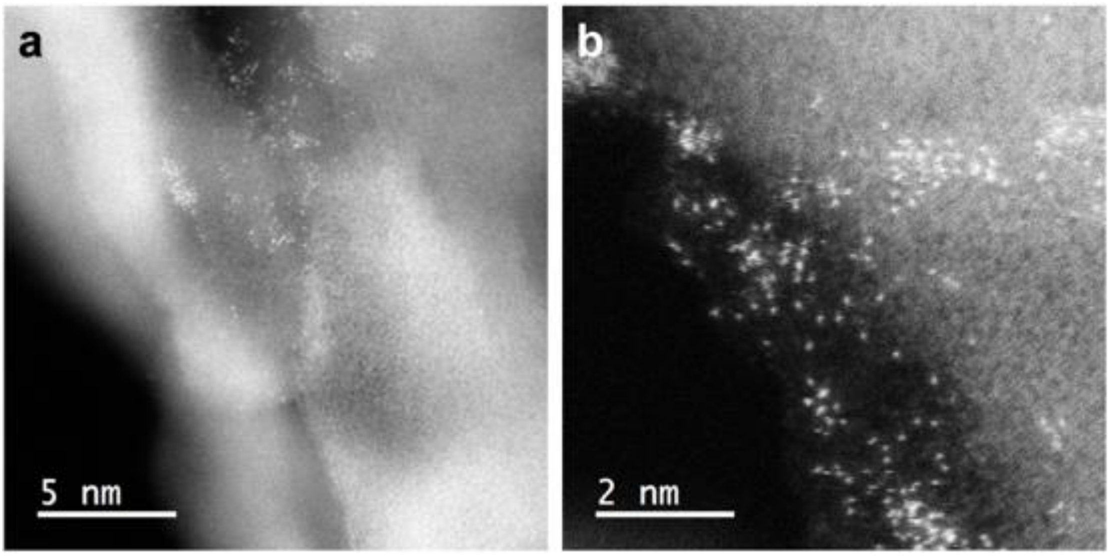


**Figure S7.** AC-HAADF-STEM images of NC/Bi SAs/TiN/CC in different regions.

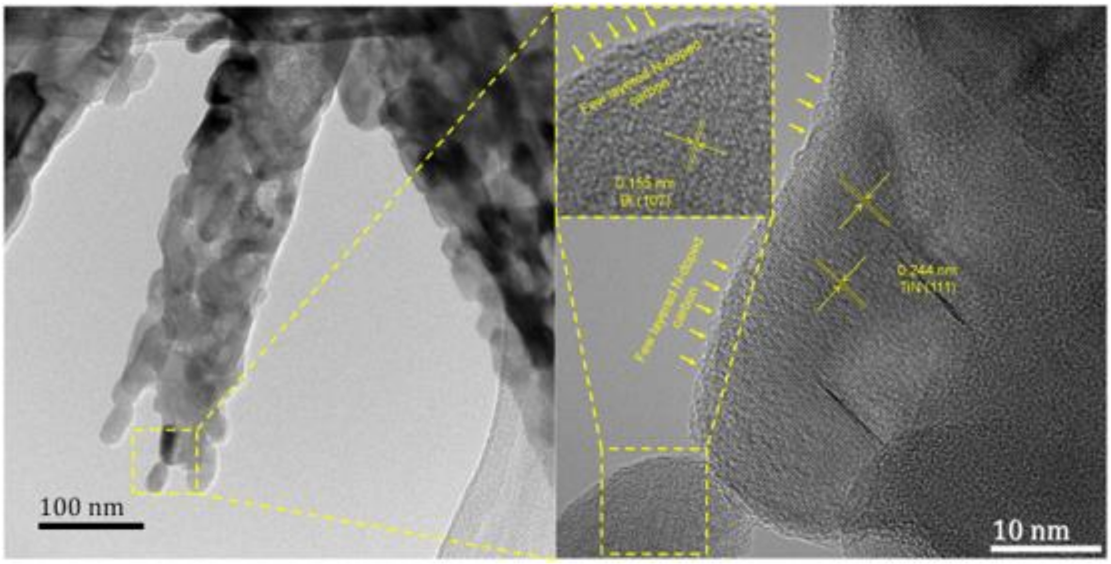


**Figure S8.** TEM images of NC/Bi NPs/TiN/CC.

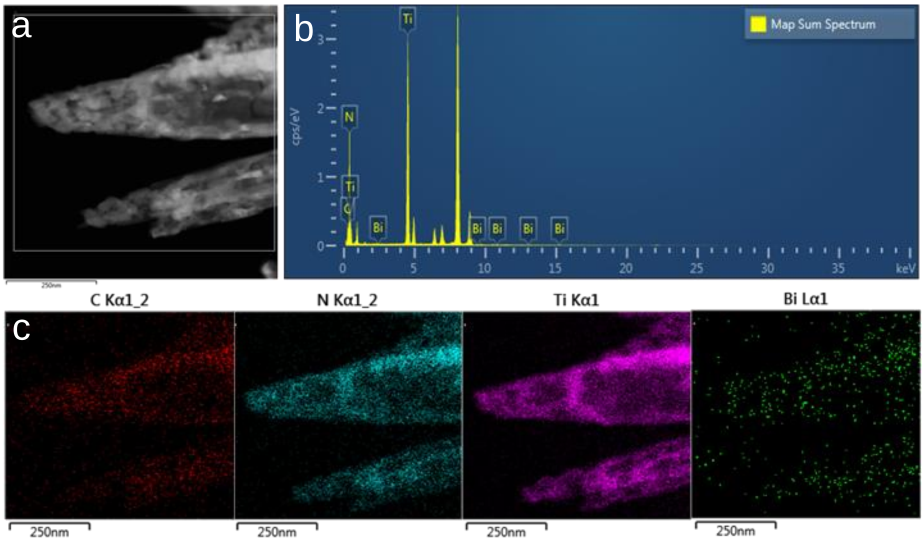


**Figure S9.** (a) STEM image and (b) EDX spectrum of NC/Bi NPs/TiN/CC. (c) The corresponding EDX elemental mappings of C, N, Ti, and Bi in NC/Bi NPs/TiN/CC.

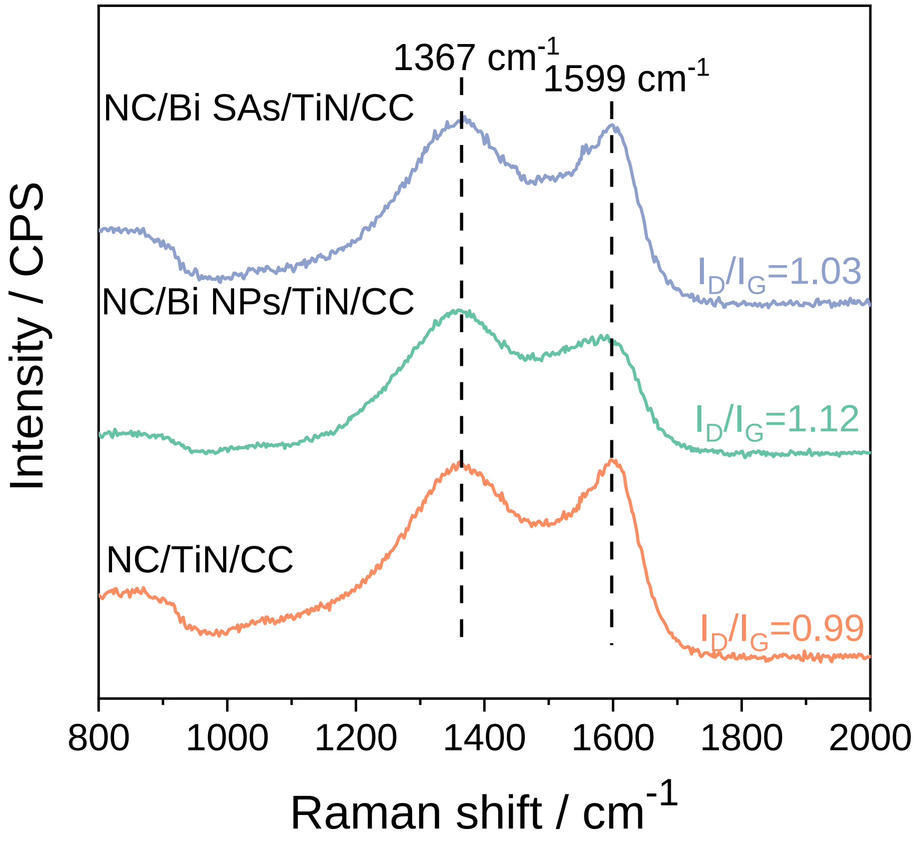


**Figure S10.** Raman spectra of NC/TiN/CC, NC/Bi SAs/TiN/CC, and NC/Bi NPs/TiN/CC.

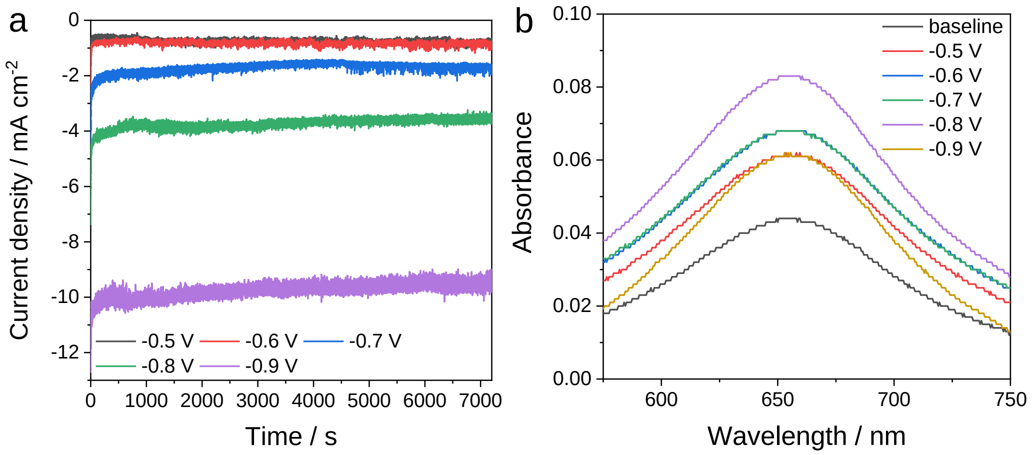


**Figure S11.** (a) Chronoamperometry curves of NC/Bi SAs/TiN/CC for ENRR at different applied potentials in $N_2$-saturated 0.1 M $Na_2SO_4$. (b) UV-vis absorption spectra of the 0.1 M $Na_2SO_4$ electrolytes after ENRR at different applied potentials for 2 h.

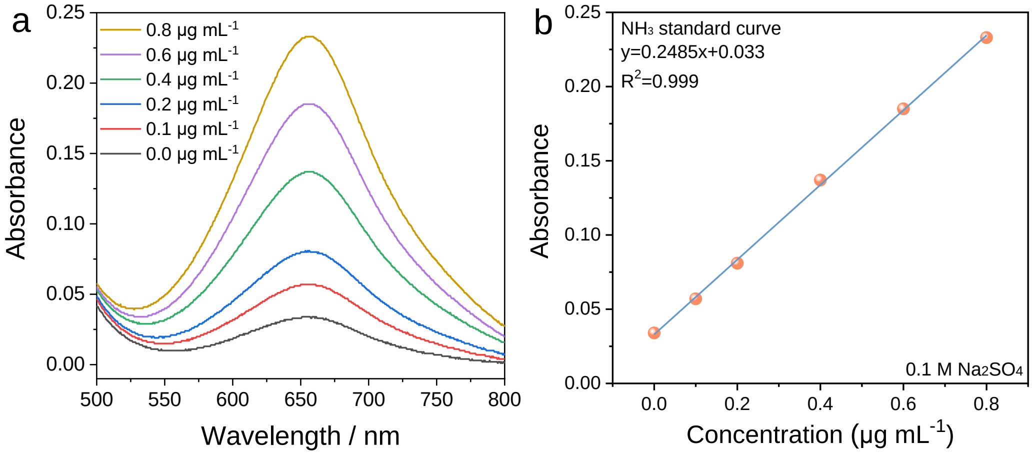


**Figure S12.** (a) UV-Vis absorption spectra of various $NH_3$ concentrations colored with indophenol blue reagent. (b) Calibration curve used for estimation of $NH_3$ concentration.

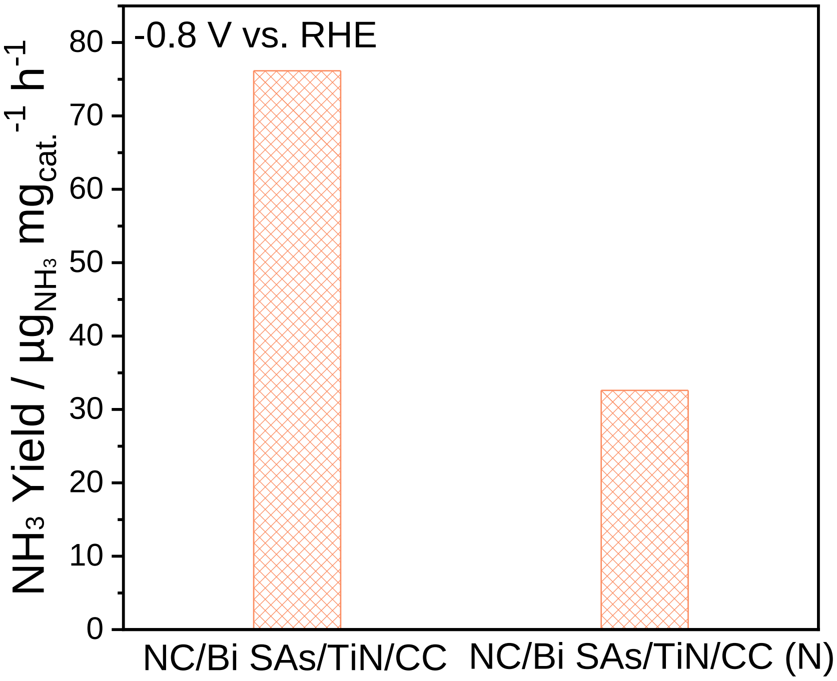


**Figure S13.** Comparison of the $NH_3$ formation rate of NC/Bi SAs/TiN/CC (N) and NC/Bi SAs/TiN/CC electrodes.

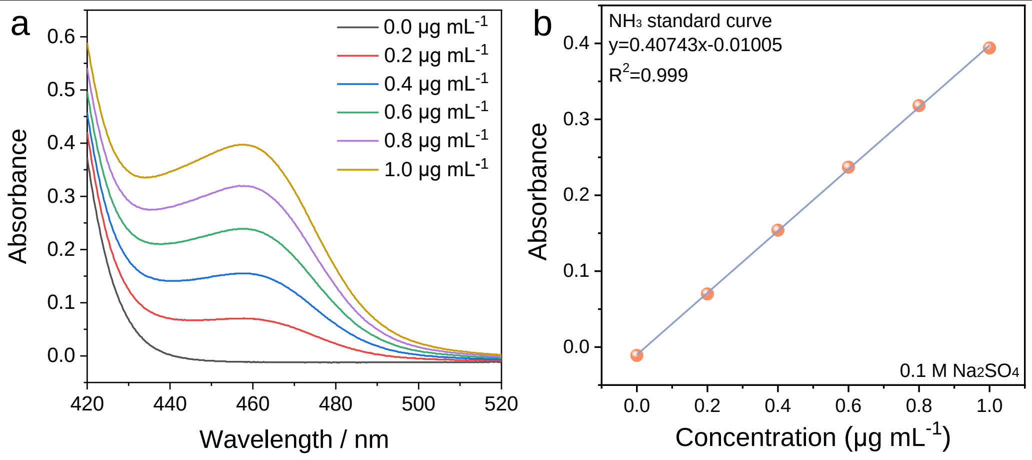


**Figure S14.** (a) UV-Vis absorption spectra of various $N_2H_4$ concentrations. (b) Calibration curve used for estimation of $N_2H_4$ concentration.

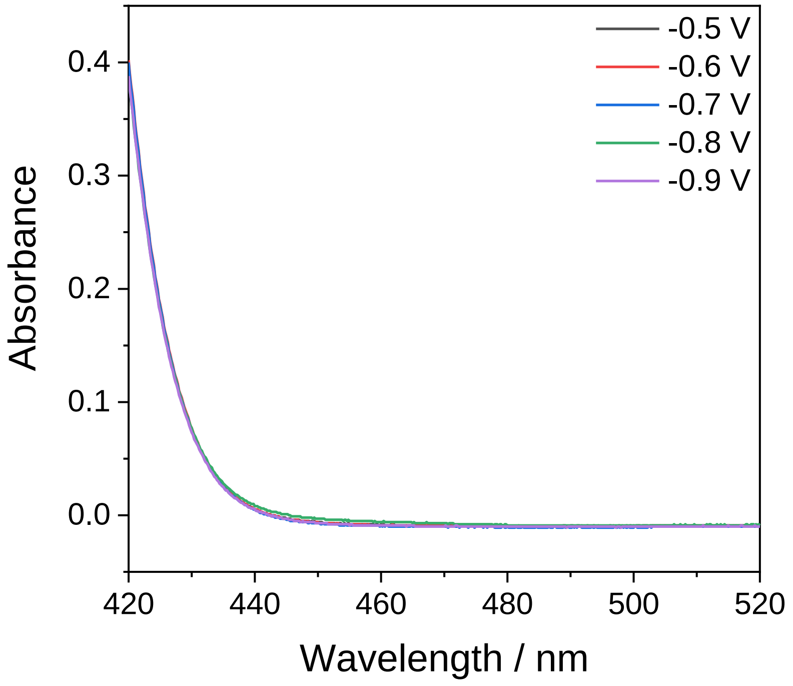


**Figure S15.** UV-vis absorption spectra of the 0.1 M $Na_2SO_4$ electrolytes after NRR at different applied potentials for 2 h by the Watt and Chrisp method.

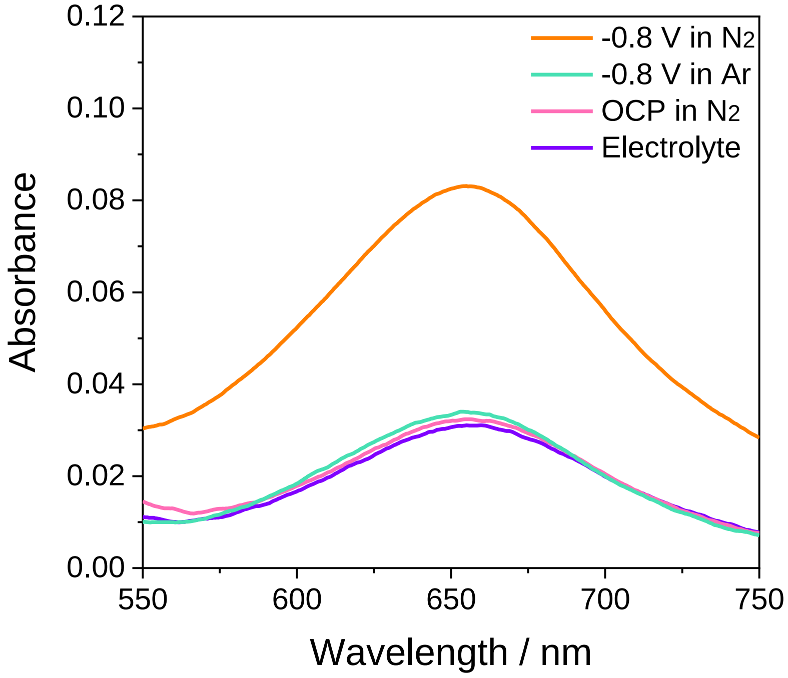


**Figure S16.** UV-Vis absorption spectra of the 0.1 M $Na_2SO_4$ electrolyte after NRR for NC/Bi SAs/TiN/CC for 2 h under different conditions.

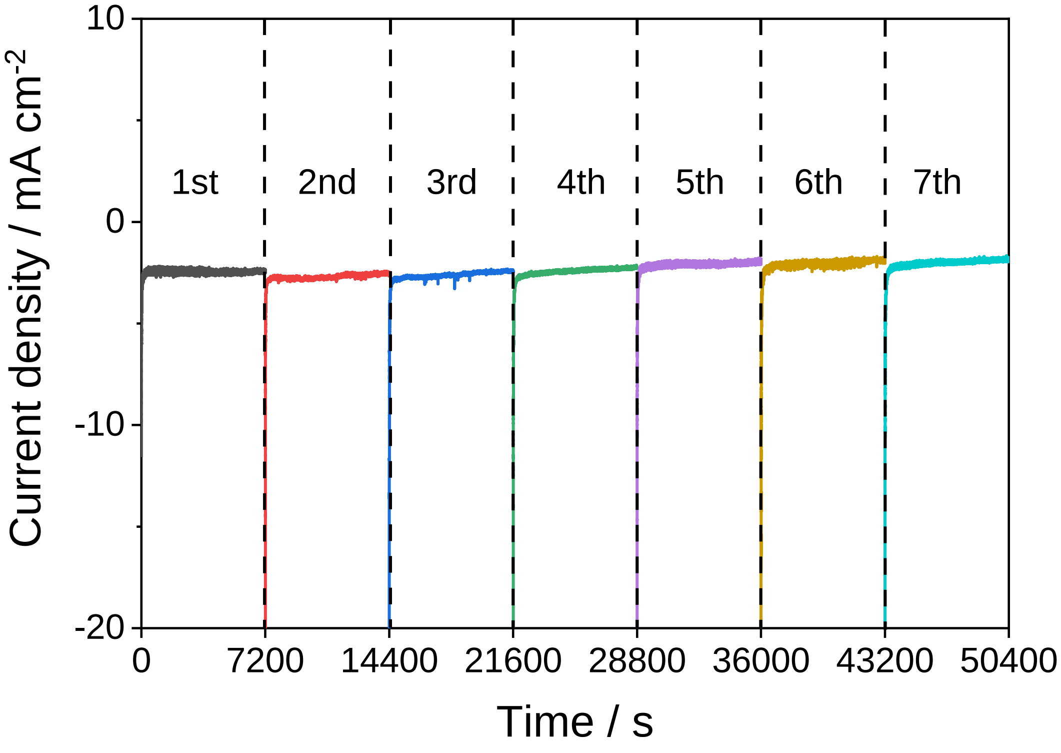


**Figure S17.** Chronoamperometry curves of NC/Bi SAs/TiN/CC for NRR at -0.8 V vs. RHE in $N_2$-saturated 0.1 M $Na_2SO_4$ with increasing cycle numbers.

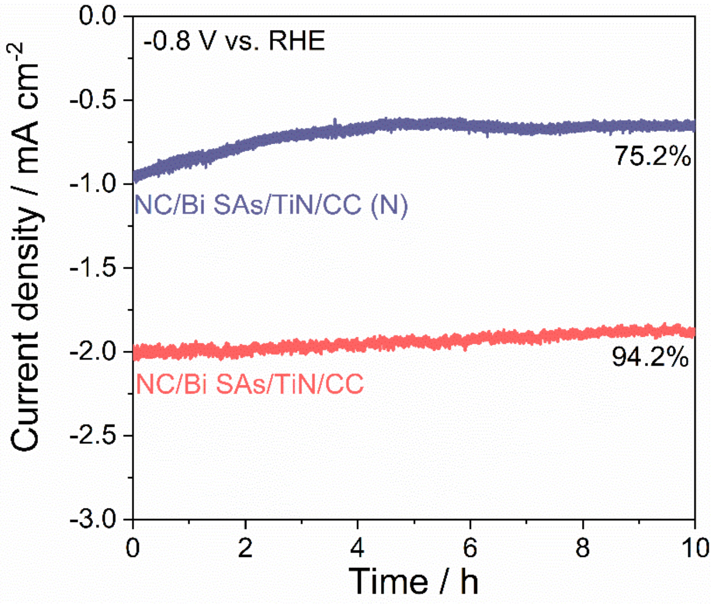


**Figure S18.** Long-term duration test for NC/Bi SAs/TiN/CC (N) and NC/Bi SAs/TiN/CC at -0.8 V (vs. RHE) in 0.1 M $Na_2SO_4$ for 10 h.

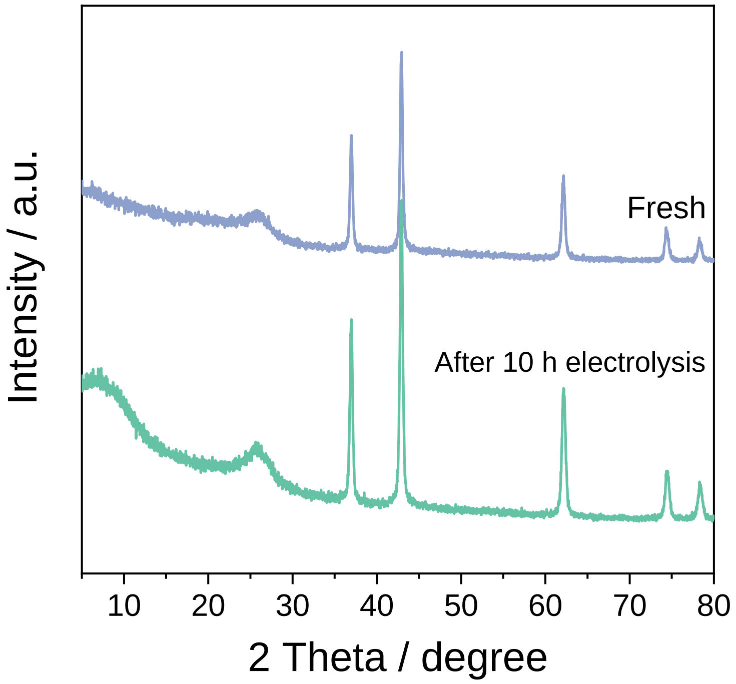


**Figure S19.** XRD patterns of fresh NC/Bi SAs/TiN/CC and NC/Bi SAs/TiN/CC after 10 h electrolysis in 0.1 M $Na_2SO_4$ electrolyte.

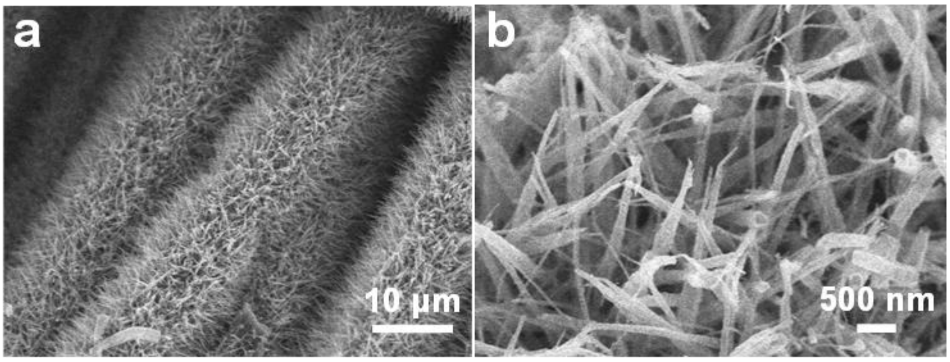


**Figure S20.** SEM images of NC/Bi SAs/TiN/CC after 10 h electrolysis in 0.1 M $Na_2SO_4$ electrolyte.

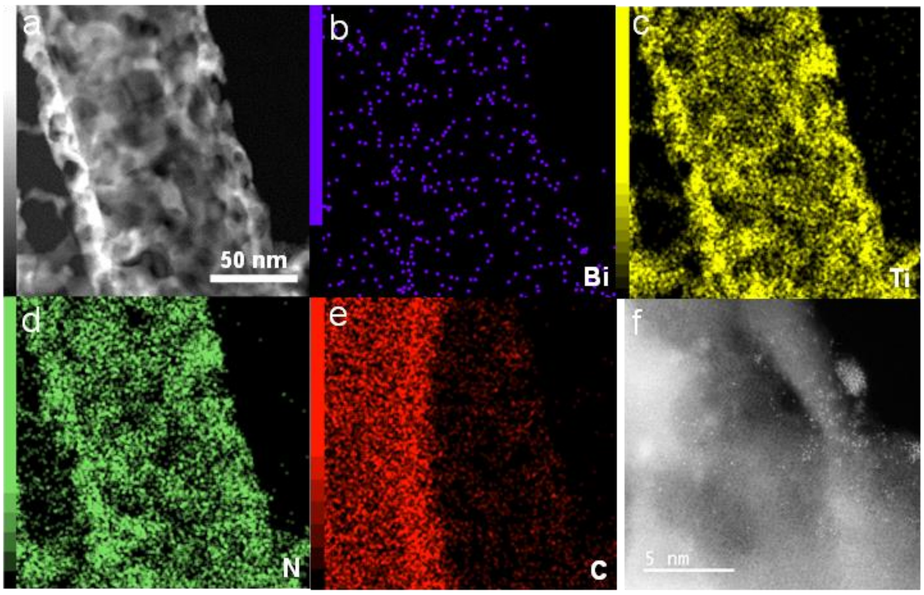


**Figure S21.** (a) STEM image of NC@Bi SAs/TiN/CC after 10 h electrolysis in 0.1 M $Na_2SO_4$ electrolyte; (b-e) Corresponding elemental mappings of Bi, Ti, N, and C in NC@Bi SAs/TiN/CC after 10 h electrolysis in 0.1 M $Na_2SO_4$ electrolyte; (f) AC-HAADF-STEM image of after 10 h electrolysis in 0.1 M $Na_2SO_4$ electrolyte.

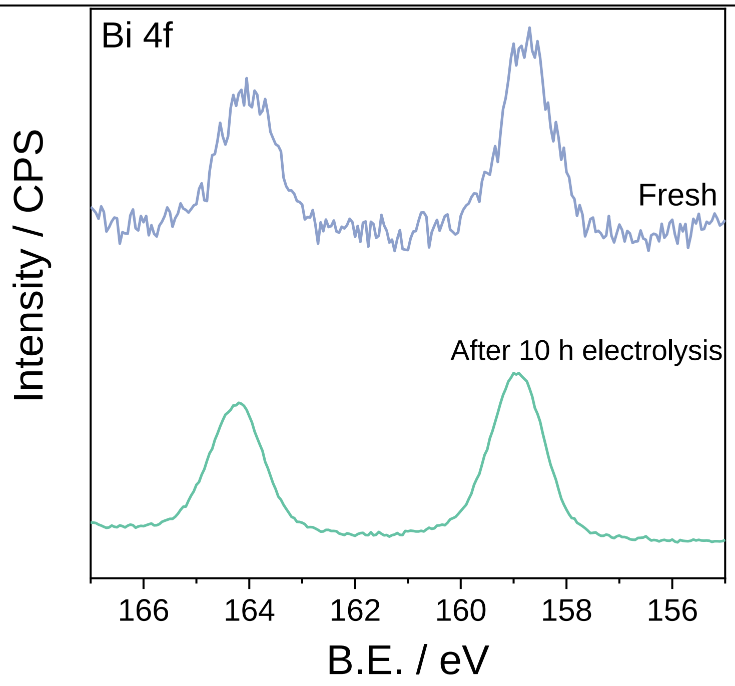


**Figure S22.** XPS spectrum of Bi 4f region in fresh NC/Bi SAs/TiN/CC and NC/Bi SAs/TiN/CC after 10 h electrolysis in 0.1 M $Na_2SO_4$ electrolyte.

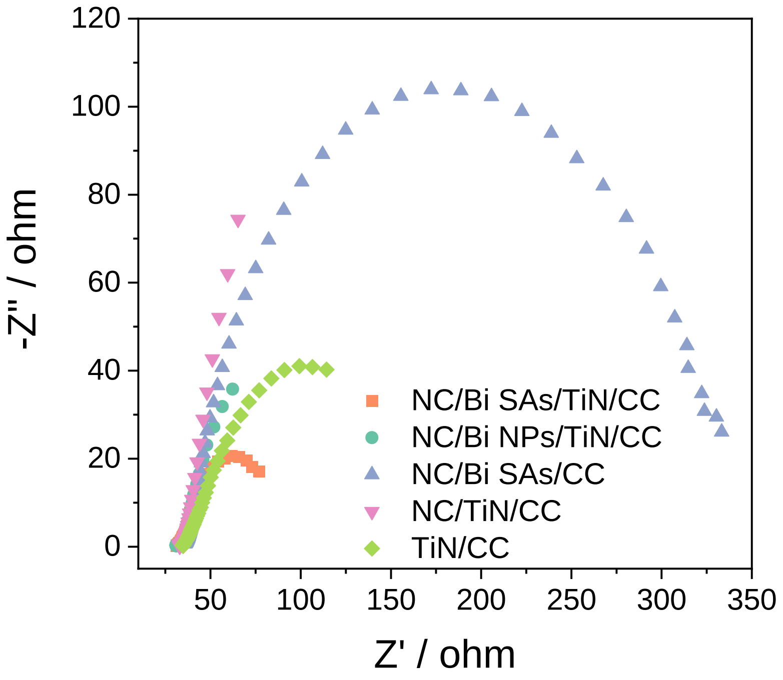


**Figure S23.** Nyquist plots of NC/Bi SAs/TiN/CC, NC/Bi NPs/TiN/CC, NC/Bi SAs/CC, NC/TiN/CC, and TiN/CC in 0.1 M $Na_2SO_4$ electrolyte.

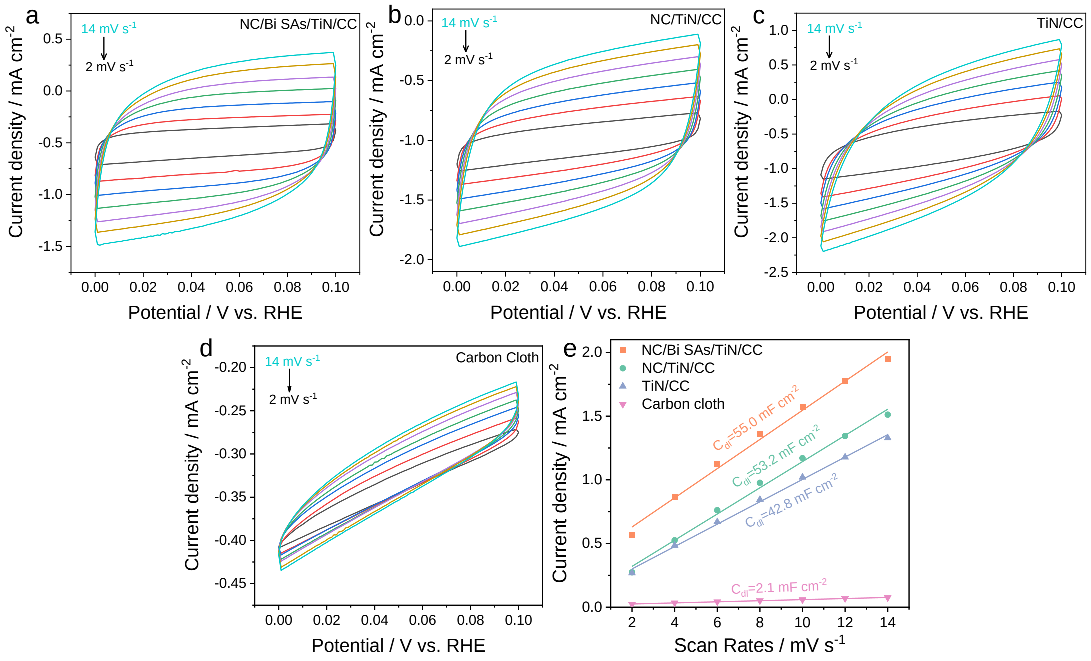


**Figure S24.** (a-d) CVs performed at various scan rates in the region 0.0–0.1 V vs. RHE and (e) Plots showing extraction of the double-layer capacitance ($C_{dl}$) for NC/Bi SAs/TiN/CC, NC/TiN/CC, TiN/CC, and carbon cloth.

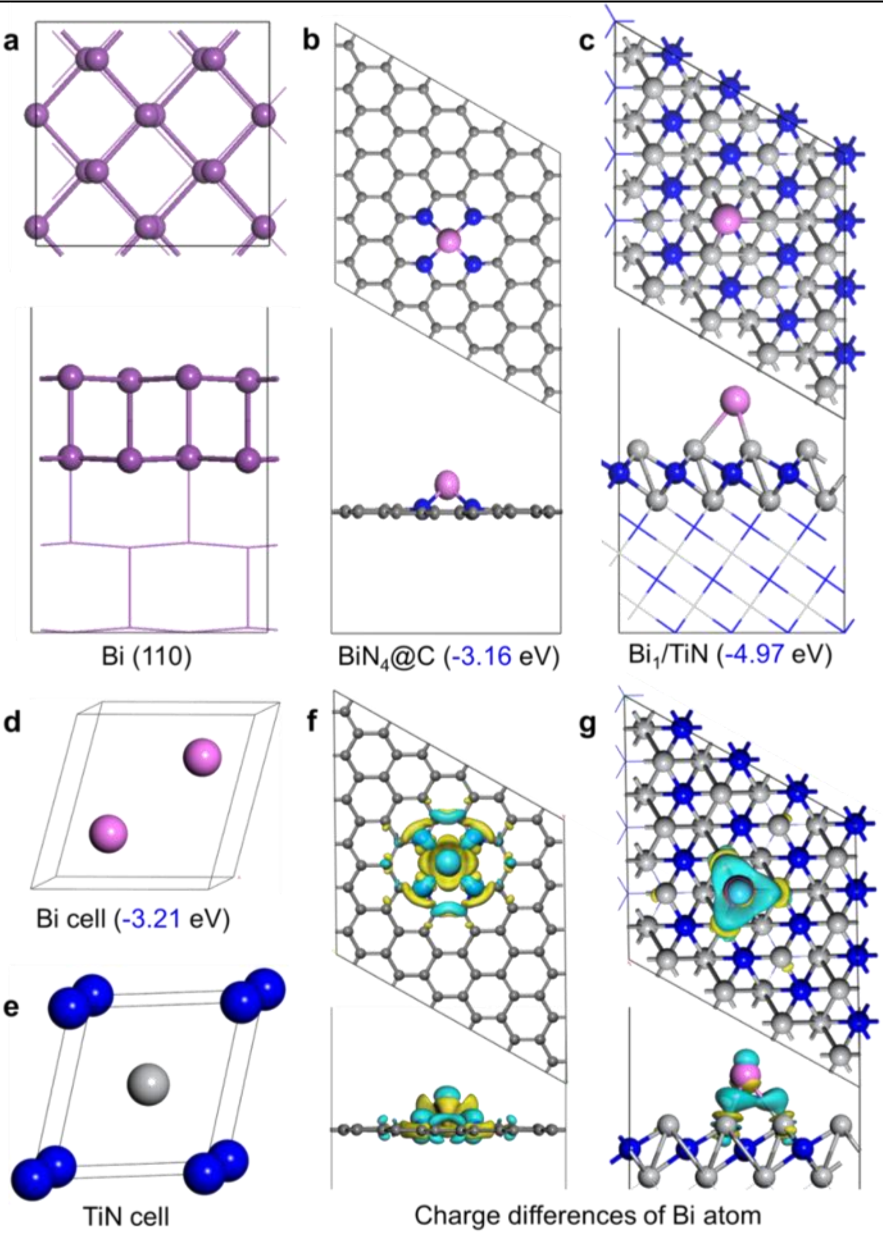


**Figure S25.** The top and side view structures of Bi (110) (**a**), $BiN_4$@C (**b**) and $Bi_1$/TiN (**c**) models, the cell structures of pure Bi (**d**) and TiN (**e**), in which the binding energies of Bi atom in the $BiN_4$@C, $Bi_1$/TiN and Bi cell are given with blue font in the bracket; the Bi, Ti, N and C atoms in the structures are shown in light pink, light gray, dark blue and gray, while the Bi atoms in Bi (110) are shown in purple. The charge differences of Bi atom in the models of $BiN_4$@C (**f**) and $Bi_1$/TiN (**g**), the cyan and yellow areas represent negative and positive charge, respectively; the cutoff of iso-surfaces is 0.1 electrons $Å^{-3}$.

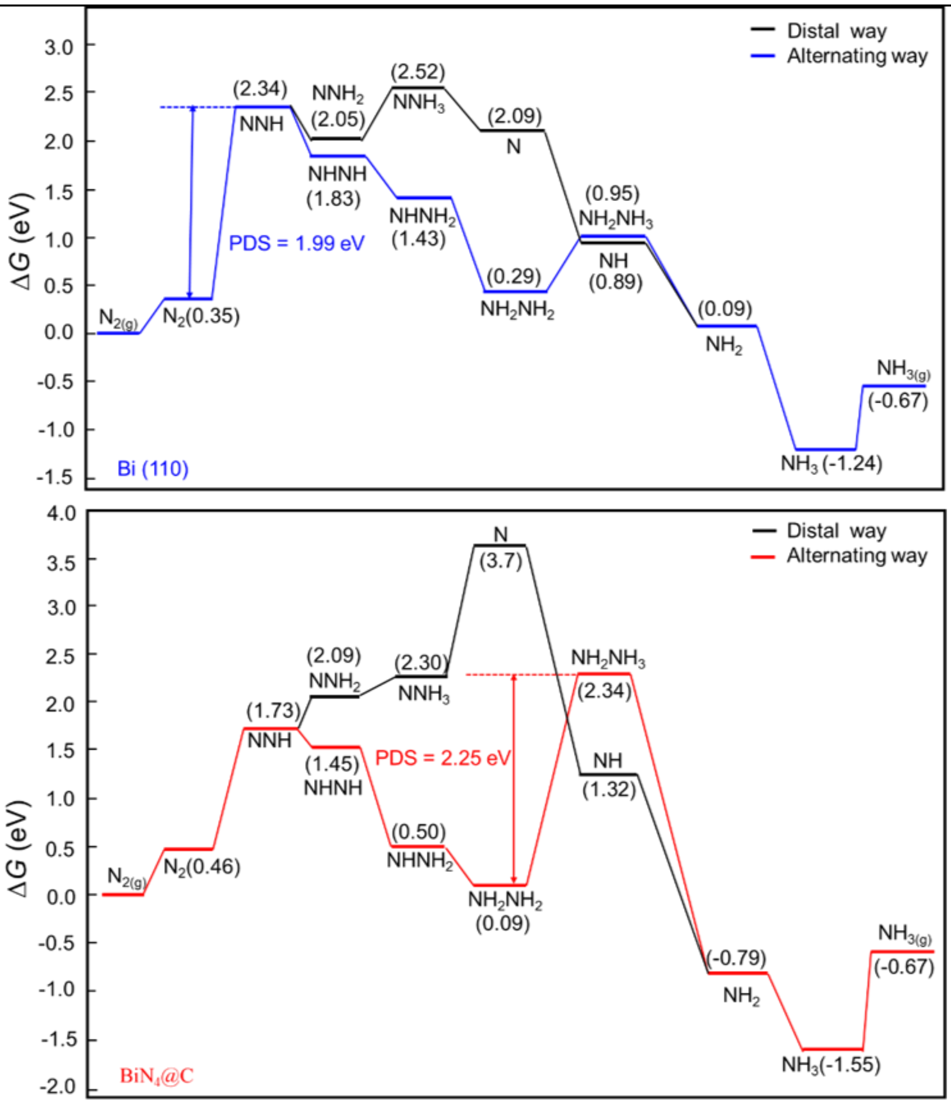


**Figure S26.** The free-energy diagrams of electrocatalytic NRR on Bi (110) and $BiN_4$@C at the electrode potential of 0V, in which the dark blue and red line represents the favorable pathway of NRR on these two models.

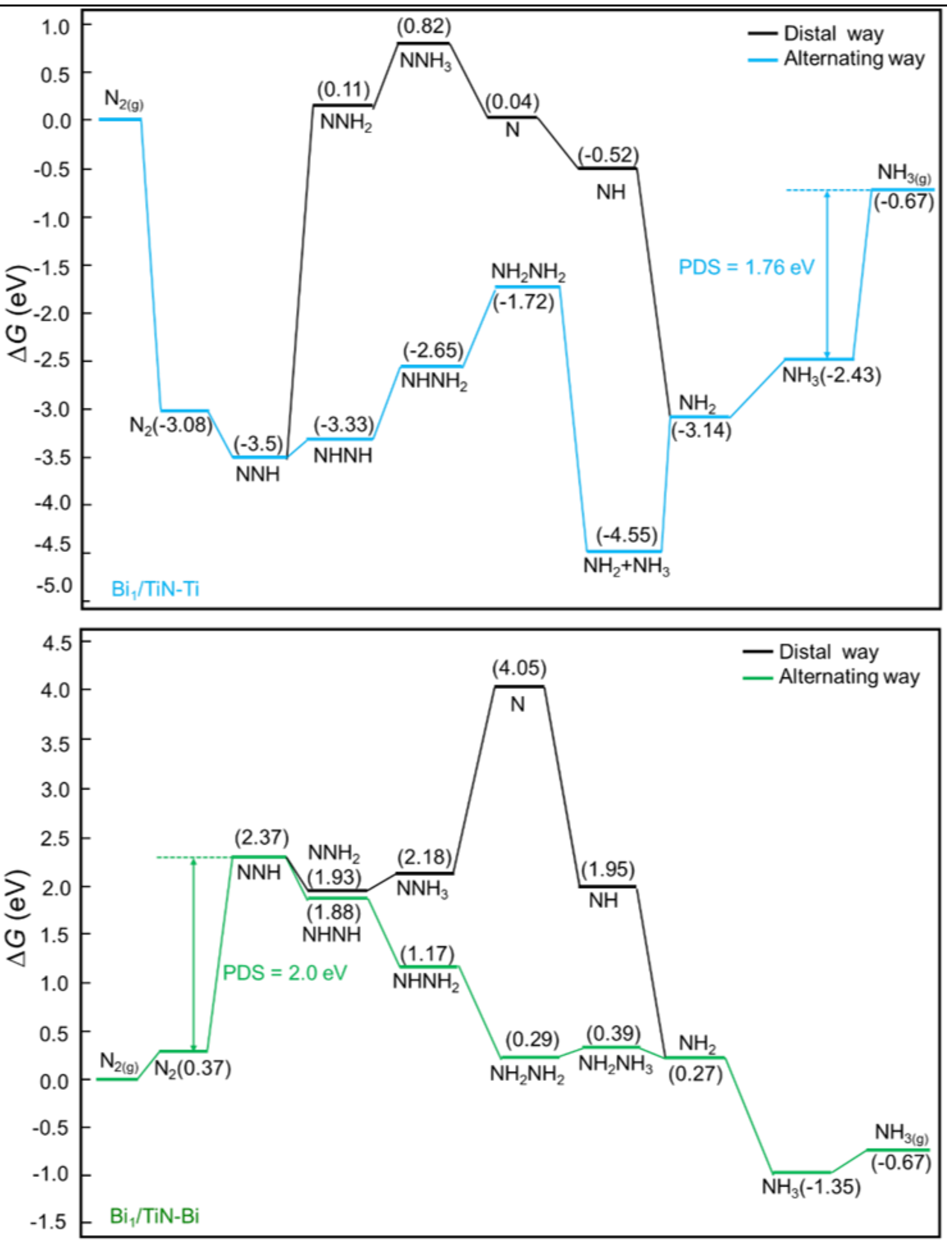


**Figure S27.** The free-energy diagrams of electrocatalytic NRR on different two active sites of Ti and Bi in the $Bi_1/TiN$ model at the electrode potential of 0V, in which the light blue and green line represents the favorable pathway of NRR on these two active sites.

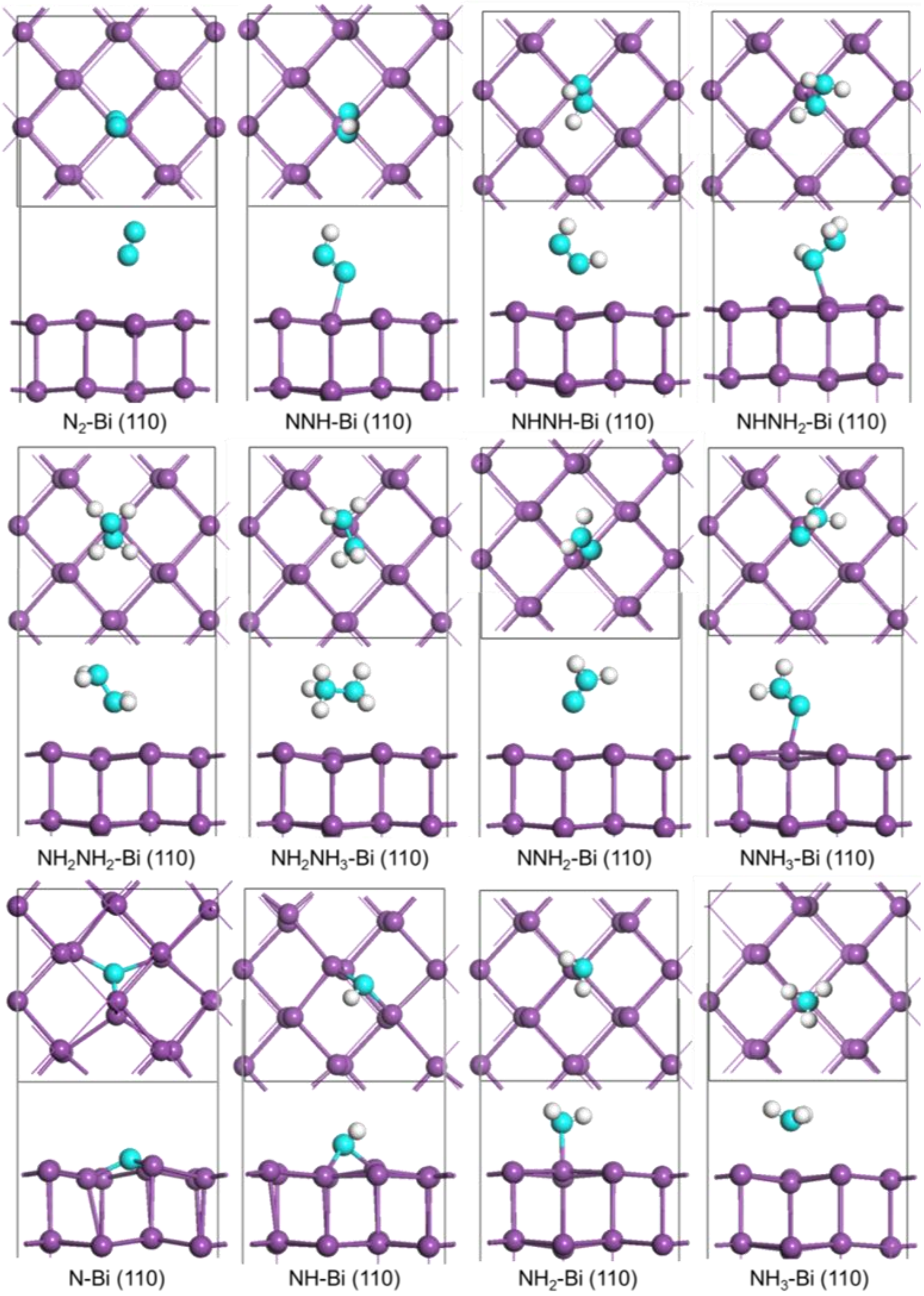


**Figure S28.** The top and side view structures of the electrocatalytic NRR intermediates on the Bi (110), the Bi, N, and H atoms are shown in purple, cyan and white, respectively.

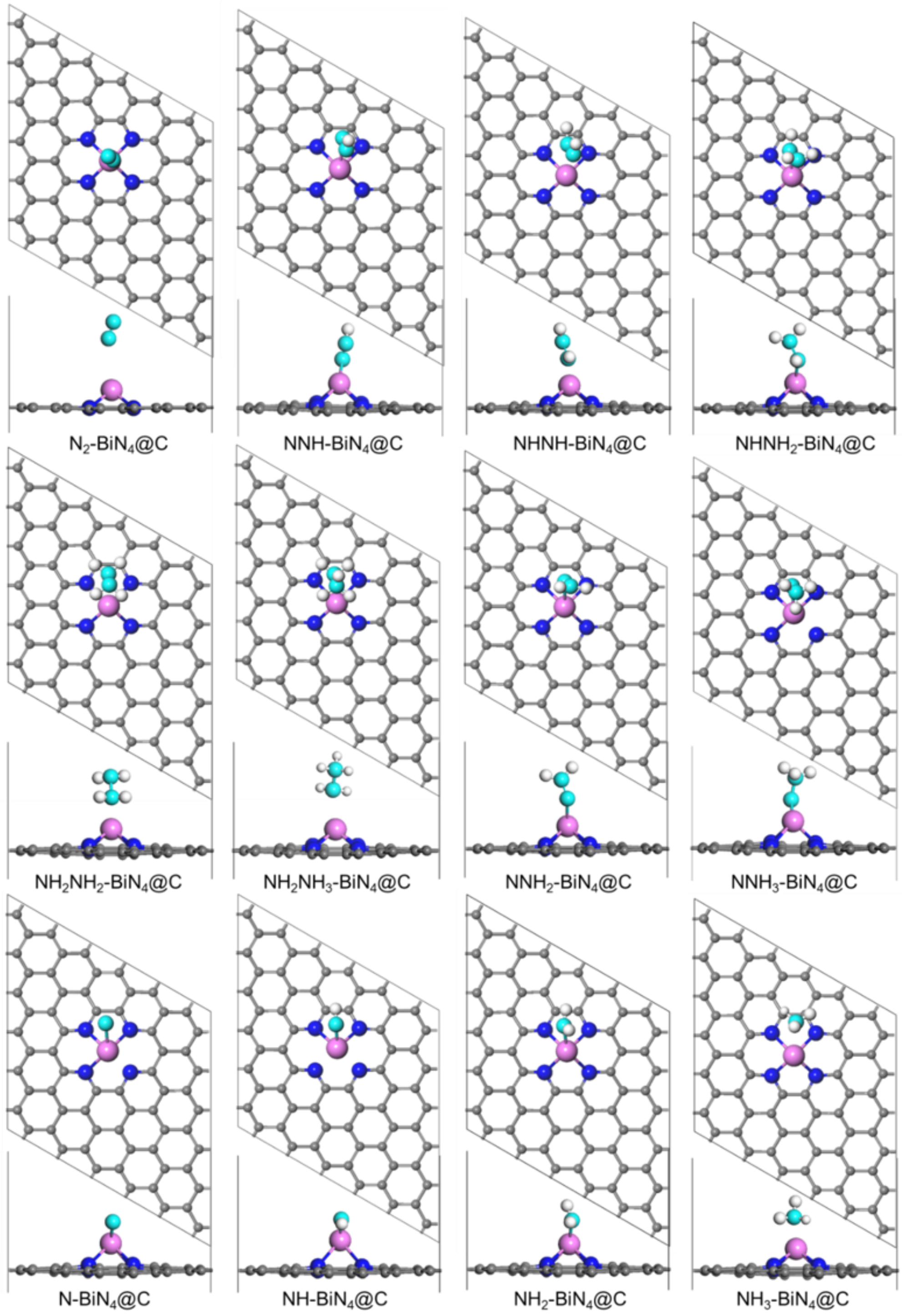


**Figure S29.** The top and side view structures of the electrocatalytic NRR intermediates on the $BiN_4$@C, the Bi, N, C and H atoms are shown in light pink, dark blue, gray, and white; to make a distinction, the N atoms from $N_2$ molecule are shown in cyan.

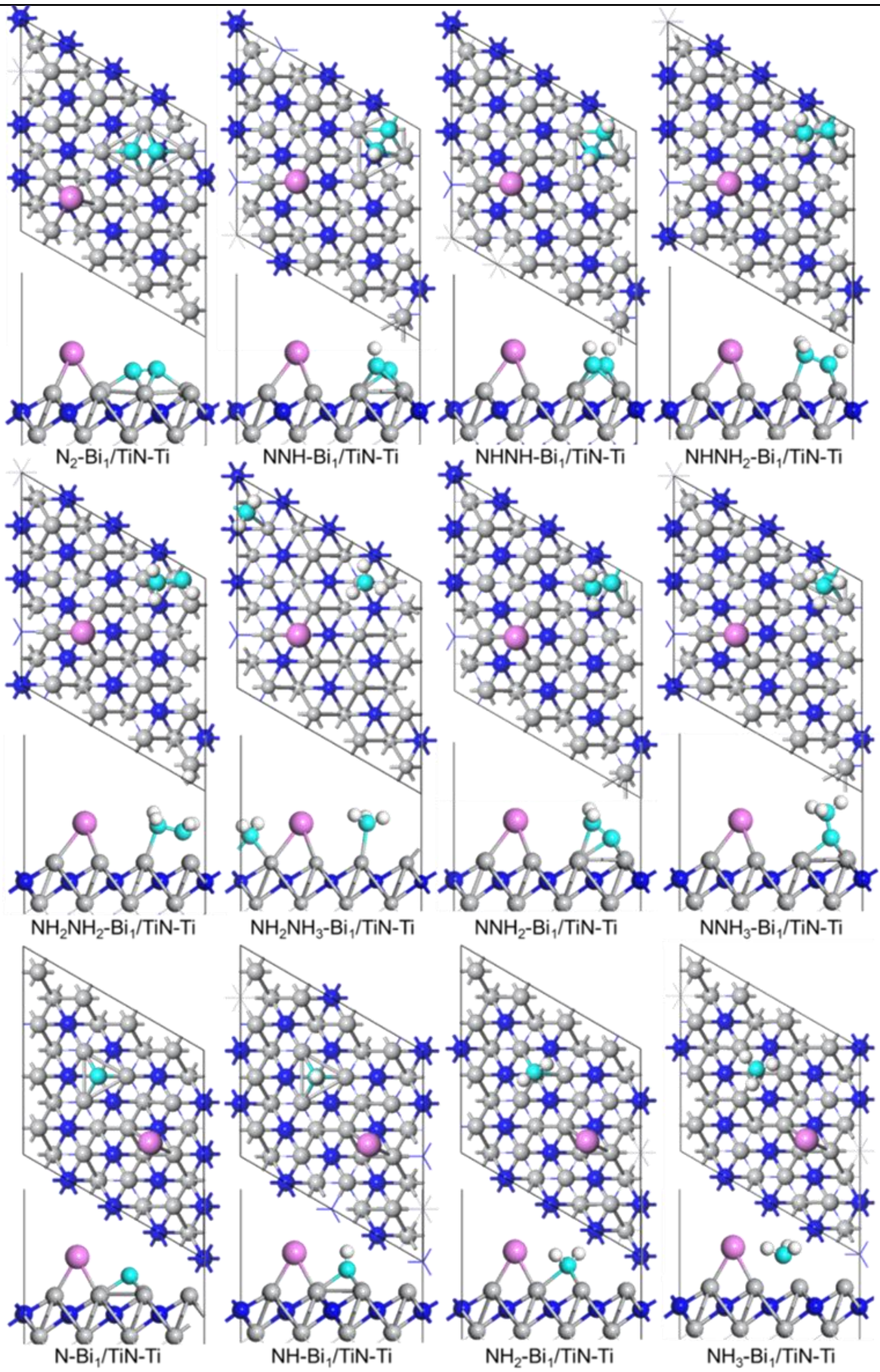


**Figure S30.** The top and side view structures of the electrocatalytic NRR intermediates on the Ti site of $Bi_1$/TiN, the Bi, Ti, N, and H atoms are shown in light pink, light gray, dark blue, and white; to make a distinction, the N atoms from $N_2$ molecule are shown in cyan.

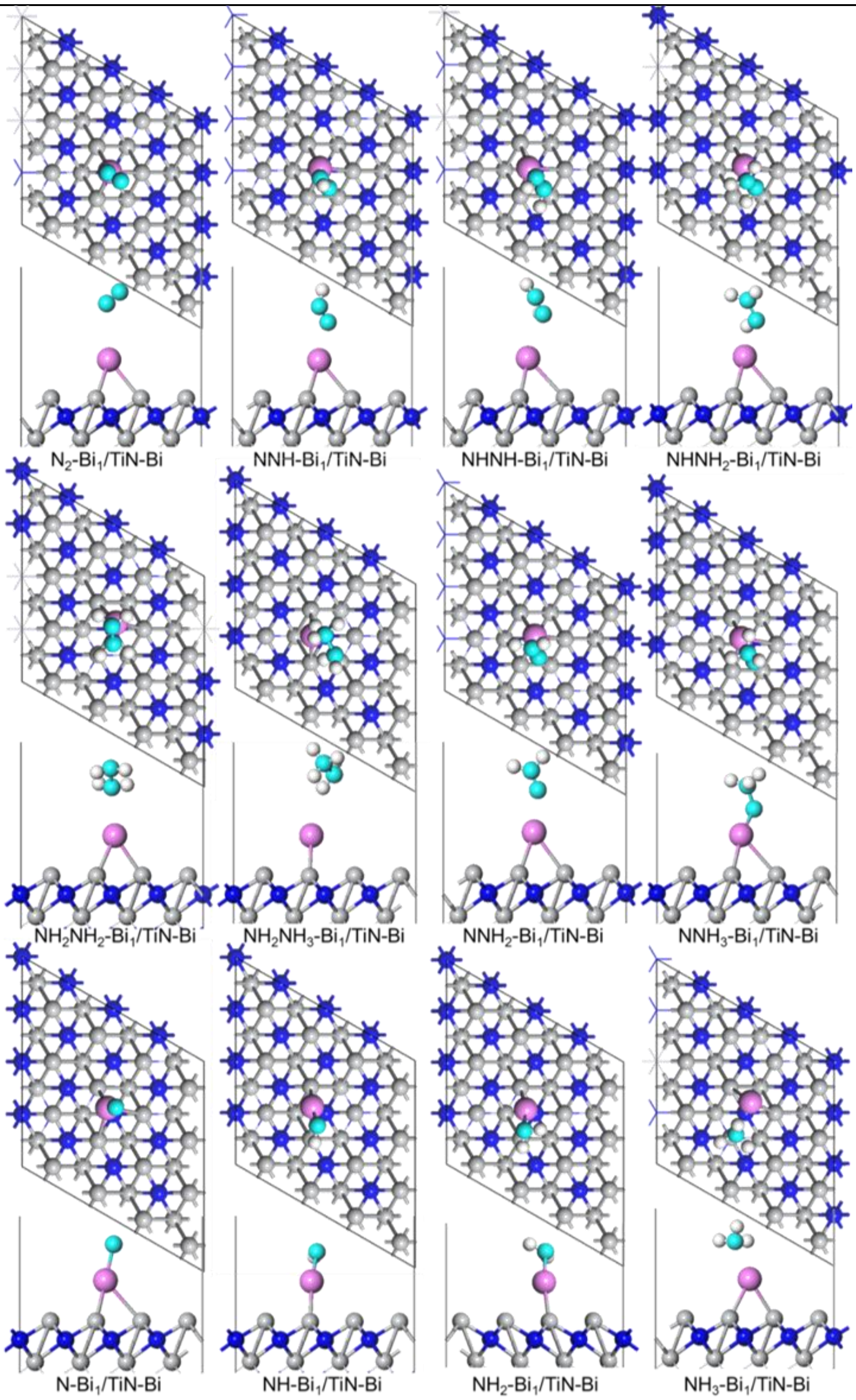


**Figure S31.** The top and side view structures of the electrocatalytic NRR intermediates on the Bi site of $Bi_1$/TiN, the Bi, Ti, N, and H atoms are shown in light pink, light gray, dark blue, and white; to make a distinction, the N atoms from $N_2$ molecule are shown in cyan.

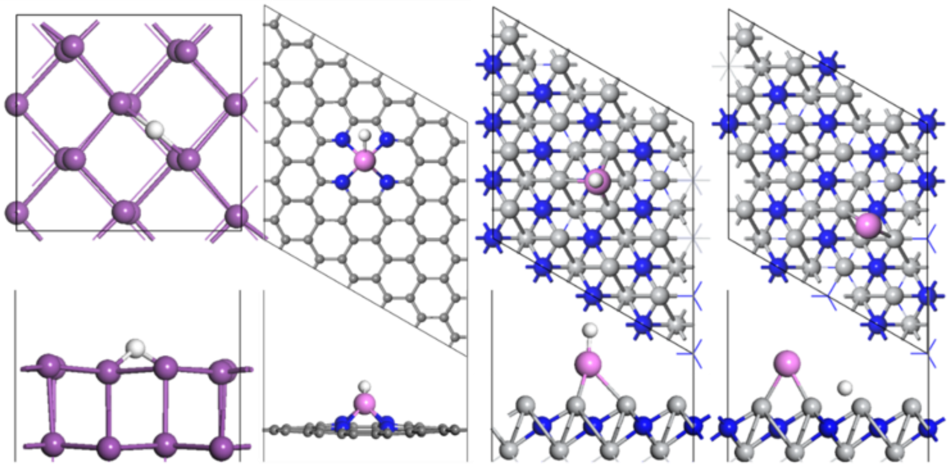


**Figure S32.** the top and side view structures of the H adsorption on the Bi (110), $BiN_4$@C, as well as the Bi and Ti site of $Bi_1$/TiN, the Bi, Ti, N, and C atoms are shown in purple, light gray, dark blue and gray; to make a distinction, the Bi single atom in the $BiN_4$@C and $Bi_1$/TiN are shown in light pink.

**Section 4. Supplementary References**

[S1] G. Kresse, J. Furthmiiller, *Comput. Mater. Sci.* **1996**, *6*, 15.

[S2] G. Kresse, J. Furthmu¨ller, *Phys. Rev. B* **1996**, *54*, 11169.

[S3] P. E. Blochl, *Phys Rev B Condens Matter* **1994**, *50*, 17953.

[S4] G. Kresse, D. Joubert, *Phys. Rev. B* **1999**, *59*, 1758.

[S5] John P. Perdew, Kieron Burke, M. Ernzerhof, *Phys. Rev. Lett.* **1996**, *77*, 3865.

[S6] W. Zang, T. Yang, H. Zou, S. Xi, H. Zhang, X. Liu, Z. Kou, Y. Du, Y. P. Feng, L. Shen, L. Duan, J. Wang, S. J. Pennycook, *ACS Catal.* **2019**, *9*, 10166.

[S7] J. Liu, X. Kong, L. Zheng, X. Guo, X. Liu, J. Shui, *ACS Nano* **2020**, *14*, 1093.

[S8] S. Zhang, M. Jin, T. Shi, M. Han, Q. Sun, Y. Lin, Z. Ding, L. R. Zheng, G. Wang, Y. Zhang, H. Zhang, H. Zhao, *Angew. Chem., Int. Ed.* **2020**, *59*, 13423.

[S9] L. Han, X. Liu, J. Chen, R. Lin, H. Liu, F. Lu, S. Bak, Z. Liang, S. Zhao, E. Stavitski, J. Luo, R. R. Adzic, H. L. Xin, *Angew. Chem., Int. Ed.* **2019**, *58*, 2321.

[S10] R. Zhang, L. Jiao, W. Yang, G. Wan, H.-L. Jiang, *J. Mater. Chem. A* **2019**, *7*, 26371.

[S11] C. He, Z.-Y. Wu, L. Zhao, M. Ming, Y. Zhang, Y. Yi, J.-S. Hu, *ACS Catal.* **2019**, *9*, 7311.

[S12] R. Hao, W. Sun, Q. Liu, X. Liu, J. Chen, X. Lv, W. Li, Y. P. Liu, Z. Shen, *Small* **2020**, *16*, 2000015.

[S13] Y. Ma, T. Yang, H. Zou, W. Zang, Z. Kou, L. Mao, Y. Feng, L. Shen, S. J. Pennycook, L. Duan, X. Li, J. Wang, *Adv. Mater.* **2020**, *32*, 2002177.

[S14] K. Chu, Q.-q. Li, Y.-p. Liu, J. Wang, Y.-h. Cheng, *Appl. Catal., B* **2020**, *267*.

[S15] C. Zhao, S. Zhang, M. Han, X. Zhang, Y. Liu, W. Li, C. Chen, G. Wang, H. Zhang, H. Zhao, *ACS Energy Lett.* **2019**, *4*, 377.

[S16] S. Mukherjee, D. A. Cullen, S. Karakalos, K. Liu, H. Zhang, S. Zhao, H. Xu, K. L. More, G. Wang, G. Wu, *Nano Energy* **2018**, *48*, 217.

[S17] X. Zhang, T. Wu, H. Wang, R. Zhao, H. Chen, T. Wang, P. Wei, Y. Luo, Y. Zhang, X. Sun, *ACS Catal.* **2019**, *9*, 4609.

[S18] Q. Liu, X. Zhang, J. Wang, Y. Zhang, S. Bian, Z. Cheng, N. Kang, H. Huang, S. Gu, Y. Wang, D. Liu, P. K. Chu, X. F. Yu, *Angew. Chem., Int. Ed.* 2020, 59, 14383.

[S19] Y. Guo, Z. Yao, B. J. J. Timmer, X. Sheng, L. Fan, Y. Li, F. Zhang, L. Sun, *Nano Energy* **2019**, *62*, 282.

[S20] Z. Zhao, S. Luo, P. Ma, Y. Luo, W. Wu, Y. Long, J. Ma, *ACS Sustainable Chem. Eng.* **2020**, *8*, 8814.

[S21] H. Jin, L. Li, X. Liu, C. Tang, W. Xu, S. Chen, L. Song, Y. Zheng, S. Z. Qiao, *Adv. Mater.* **2019**, *31*, 1902709.

[S22] N. Zhang, F. Zheng, B. Huang, Y. Ji, Q. Shao, Y. Li, X. Xiao, X. Huang, *Adv. Mater.* **2020**, *32*, 1906477.

[S23] T. Wu, H. Zhao, X. Zhu, Z. Xing, Q. Liu, T. Liu, S. Gao, S. Lu, G. Chen, A. M. Asiri, Y. Zhang, X. Sun, *Adv. Mater.* **2020**, *32*, 2000299.

[S24] Z. Jin, C. Liu, Z. Liu, J. Han, Y. Fang, Y. Han, Y. Niu, Y. Wu, C. Sun, Y. Xu, *Adv. Energy Mater.* **2020**, *10*.

[S25] W. Xu, G. Fan, J. Chen, J. Li, L. Zhang, S. Zhu, X. Su, F. Cheng, J. Chen, *Angew.*

*Chem., Int. Ed.* **2020**, *59*, 3511.

[S26] Y. Zhang, J. Hu, C. Zhang, Y. Liu, M. Xu, Y. Xue, L. Liu, M. K. H. Leung, *J. Mater. Chem. A* **2020**, *8*, 9091.

[S27] P. Wei, Q. Geng, A. I. Channa, X. Tong, Y. Luo, S. Lu, G. Chen, S. Gao, Z. Wang, X. Sun, *Nano Res.* **2020**, *13*, 2967.

[S28] Y.-C. Hao, Y. Guo, L.-W. Chen, M. Shu, X.-Y. Wang, T.-A. Bu, W.-Y. Gao, N. Zhang, X. Su, X. Feng, J.-W. Zhou, B. Wang, C.-W. Hu, A.-X. Yin, R. Si, Y.-W. Zhang, C.-H. Yan, *Nat. Catal.* **2019**, *2*, 448.

[S29] Y. Wan, H. Zhou, M. Zheng, Z. H. Huang, F. Kang, J. Li, R. Lv, *Adv. Funct. Mater.* **2021**, *31*, 2100300.

[S30] F. Xu, F. Wu, K. Zhu, Z. Fang, D. Jia, Y. Wang, G. Jia, J. Low, W. Ye, Z. Sun, P. Gao, Y. Xiong, *Appl. Catal., B* **2021**, *284*, 119689.

[S31] L. Zhao, J. Zhou, L. Zhang, X. Sun, X. Sun, T. Yan, X. Ren, Q. Wei, *ACS Appl. Mater. Interfaces* **2020**, *12*, 55838.

[S32] Y. Qiu, S. Zhao, M. Qin, J. Diao, S. Liu, L. Dai, W. Zhang, X. Guo, *Inorganic Chemistry Frontiers* **2020**, *7*, 2006.

[S33] F. Wang, X. Lv, X. Zhu, J. Du, S. Lu, A. A. Alshehri, K. A. Alzahrani, B. Zheng, X. Sun, *Chem. Commun.* **2020**, *56*, 2107.

[S34] C. Lv, C. Yan, G. Chen, Y. Ding, J. Sun, Y. Zhou, G. Yu, *Angew. Chem., Int. Ed.* **2018**, *57*, 6073.

[S35] Y. Xu, T. Ren, S. Yu, K. Ren, M. Wang, Z. Wang, X. Li, L. Wang, H. Wang, *Sustainable Energy & Fuels* **2020**, *4*, 4516.

[S36] B. Chang, Q. Liu, N. Chen, Y. Yang, *ChemCatChem* **2019**, *11*, 1884.

[S37] L. Li, C. Tang, B. Xia, H. Jin, Y. Zheng, S.-Z. Qiao, *ACS Catal.* **2019**, *9*, 2902.

[S38] Y. Xu, T. Ren, S. Yu, H. Yu, S. Yin, Z. Wang, X. Li, L. Wang, H. Wang, *Sustainable Energy Fuels* **2020**, *4*, 3334.

[S39] L. Xia, W. Fu, P. Zhuang, Y. Cao, M. O. L. Chee, P. Dong, M. Ye, J. Shen, *ACS Sustainable Chem. Eng.* **2020**, *8*, 2735.

[S40] Y. Liu, C. Li, L. Guan, K. Li, Y. Lin, *J. Phys. Chem. C* **2020**, *124*, 18003.

[S41] D. Yao, C. Tang, L. Li, B. Xia, A. Vasileff, H. Jin, Y. Zhang, S. Z. Qiao, *Adv. Energy Mater.* **2020**, *10*, 2001289.

[S42] J. X. Yao, D. Bao, Q. Zhang, M. M. Shi, Y. Wang, R. Gao, J. M. Yan, Q. Jiang, *Small Methods* **2018**, *3*, 1800333.

[S43] Y. Wang, M. M. Shi, D. Bao, F. L. Meng, Q. Zhang, Y. T. Zhou, K. H. Liu, Y. Zhang, J. Z. Wang, Z. W. Chen, D. P. Liu, Z. Jiang, M. Luo, L. Gu, Q. H. Zhang, X. Z. Cao, Y. Yao, M. H. Shao, Y. Zhang, X. B. Zhang, J. G. Chen, J. M. Yan, Q. Jiang, *Angew. Chem., Int. Ed.* **2019**, *58*, 9464.

[S44] Y. Sun, Z. Deng, X.-M. Song, H. Li, Z. Huang, Q. Zhao, D. Feng, W. Zhang, Z. Liu, T. Ma, *Nano-Micro Lett.* **2020**, *12*, 132.

[S45] R. Zhang, L. Ji, W. Kong, H. Wang, R. Zhao, H. Chen, T. Li, B. Li, Y. Luo, X. Sun, *Chem. Commun.* **2019**, *55*, 5263.
